# The TTYH3/MK5 Positive Feedback Loop regulates Tumor Progression via GSK3-β/β-catenin signaling in HCC

**DOI:** 10.7150/ijbs.73009

**Published:** 2022-06-21

**Authors:** Yixiu Wang, Yuwei Xie, Bingzi Dong, Weijie Xue, Shuhai Chen, Shimada Mitsuo, Hao Zou, Yujie Feng, Kai Ma, Qian Dong, Jingyu Cao, Chengzhan Zhu

**Affiliations:** 1 Department of Hepatobiliary and Pancreatic Surgery, The Affiliated Hospital of Qingdao University, No.16 Jiangsu Road, Qingdao 266003, China.; 2 Shandong Key Laboratory of Digital Medicine and Computer Assisted Surgery, The Affiliated Hospital of Qingdao University, No. 16 Jiangsu Road, Qingdao 266003, China.; 3 Department of Endocrinology and Metabolism, The Affiliated Hospital of Qingdao University, No.16 Jiangsu Road, Qingdao 266003, China.; 4 Department of Gastrointestinal Surgery, The Affiliated Hospital of Qingdao University, No.16 Jiangsu Road, Qingdao 266003, China.; 5 Department of Surgery, Institute of Biomedical Sciences, Tokushima University, Tokushima 770‑8503, Japan.; 6 Department of Pediatric Surgery, The Affiliated Hospital of Qingdao University, No. 16 Jiangsu Road, Qingdao 266003, China.

**Keywords:** Tweety homolog 3, hepatocellular carcinoma, tumor metastasis, epithelial-mesenchymal transition, positive feedback

## Abstract

Hepatocellular carcinoma (HCC) is one of the leading causes of cancer-related death worldwide, and identification of novel targets is necessary for its diagnosis and treatment. This study aimed to investigate the biological function and clinical significance of tweety homolog 3 (TTYH3) in HCC. TTYH3 overexpression promoted cell proliferation, migration, and invasion and inhibited HCCM3 and Hep3B cell apoptosis. TTYH3 promoted tumor formation and metastasis *in vivo*. TTYH3 upregulated calcium influx and intracellular chloride concentration, thereby promoting cellular migration and regulating epithelial-mesenchymal transition-related protein expression. The interaction between TTYH3 and MK5 was identified through co-immunoprecipitation assays and protein docking. TTYH3 promoted the expression of MK5, which then activated the GSK3β/β-catenin signaling pathway. *MK5* knockdown attenuated the activation of GSK3β/β-catenin signaling by TTYH3. TTYH3 expression was regulated in a positive feedback manner. In clinical HCC samples, TTYH3 was upregulated in the HCC tissues compared to nontumor tissues. Furthermore, high TTYH3 expression was significantly correlated with poor patient survival. The CpG islands were hypomethylated in the promoter region of *TTYH3* in HCC tissues. In conclusion, we identified TTYH3 regulates tumor development and progression via MK5/GSK3-β/β-catenin signaling in HCC and promotes itself expression in a positive feedback loop.

## Introduction

Primary liver cancer is the third leading cause of cancer-related death worldwide 1. As China has the world's largest population, it has the greatest number of cases of primary liver cancer. Surgical resection and liver transplantation are the optimal treatment options for HCC, but early recurrence and metastasis are the main factors associated with poor prognosis 2. Although new targeted therapy drugs and immune checkpoint inhibitors have significantly improved patient survival, the median overall survival (OS) time for late-stage HCC is still less than two years 3. Thus, identifying new targets for liver cancer treatment is still highly important.

Ion channels are involved in almost all pathophysiological processes of cancer development, albeit to varying extents 4. Ion channels of calcium (Ca^2+^), sodium (Na^+^), potassium (K^+^), chloride (Cl^-^), and zinc (Zn^2+^) are involved in the biological function of gastrointestinal cancer and HCC 5-7. A survival model has been established with ion channel genes, including ANO10 and CLCN2, for HCC risk prediction 8. The mRNA expression profile in a rat liver cancer development model has shown that the ion channel genes are potential early markers or therapeutic targets of HCC 9. Ca^2+^-mediated signaling pathways are implicated in tumor metastasis and metabolism regulation 10. Moreover, several chloride channels, including CLCA4 and CLC3, are also reportedly involved in HCC progression 11. However, the biological function of ion channels in HCC still requires further study.

The human tweety homolog (TTYH) family (TTYH1-3), which comprises homologs of *tweety* in flightless Drosophila, encode large conductance chloride channels 12. TTYH family members are involved in embryonic development 13, 14, brain pathology 15, and tumor progression 16, 17. TTYH1 is mainly expressed in the brain and encodes a Ca^2+^-independent and swelling-activated Cl^-^ channel 12. TTYH1 regulates neural stem cell function 18, embryonic development 19, and promotes brain tumor formation 16, while TTYH2 and TTYH3 encode Ca^2+^-activated Cl^-^ channels 12. *TTYH2* was found to be overexpressed in renal 20 and colon cancer 17, and TTYH3 was mainly expressed in excitable tissues, including the brain, heart, and skeletal muscle 21. TTYH3 is a lager conductance Ca^2+^-activated Cl^-^ channel with complex gating kinetics and voltage-dependent inactivation. Its activation is dependent on the intracellular Ca^2+^ concentration. Similar to the maxi-Cl (-) channel, the TTYH3 channel shows linear current voltage (26-picosiemen) and an anion permeability order of I^-^ > Br^-^ > Cl^-^ 21. Bioinformatics analysis has indicated that TTYH3 is overexpressed in gastric cancer and is related to poor prognosis 22; however, its biological function and clinical significance in HCC has never been studied.

Here, we investigated the biological function of TTYH3 in HCC both *in vitro* and *in vivo*. We also examined the mechanism by which TTYH3 promoted cancer invasion and migration. Then, we confirmed TTYH3 expression in HCC tissue and analyzed its correlation with patient prognosis. DNA methylation microarray and pyrophosphate sequencing were performed to explore the possible mechanism of high TTYH3 expression in HCC tissue.

## Methods

### Cell lines and cell culture

HCC cell lines Hep3B and HCCLM3 were obtained from the Cell Resource Center of Shanghai Institutes for Biological Sciences, Chinese Academy of Sciences (Shanghai, China). In brief, cells were cultured in DMEM (Hyclone, Utah, USA) containing 10% fetal bovine serum and incubated at 37 °C in a 5% CO_2_ incubator. Cells were treated with different concentration of CaCl2 or Methyl 3-methyl {[(4-methylphenyl) sulfonyl] amino} benzoate (MSAB, MedChemExpress, Shanghai, China) for molecular function analysis.

### Patients

A total of 140 patients diagnosed with HCC in the Affiliated Hospital of Qingdao University from January 2013 to December 2018 were enrolled in the study. Moreover, a total of 58 patients diagnosed with HCC in the Institute of Biomedical Sciences, Tokushima University were enrolled in the study. All patients underwent hepatectomy for initial diagnosis of HCC. The resected tumor tissues and the corresponding adjacent nontumor liver tissues were collected and frozen at -80 °C. Pathological examination confirmed all tumors were HCC. The clinicopathological data was obtained from medical records, and the patients were followed up until December 2020. The study was approved by the Ethics Committee of the Affiliated Hospital of Qingdao University and the Institute of Biomedical Sciences, Tokushima University. Informed written consent was obtained from all patients included in the study.

### RNA sequencing (RNA-seq) and quantitative real-time PCR (qPCR) analysis

RNA-seq was performed to determine the mRNA expression level of TTYH3 in 39 HCC tissues from the Affiliated Hospital of Qingdao University. The total RNA was extracted from each specimen, prepared RNA sequence libraries with Illumina's Kapa Hyper Prep Kit, and sequenced by Illumina NovaSeq 6000 system. qPCR was performed using the SYBR Green method on a Roche 480 system to detect mRNA expression in 39 HCC tissues from the Affiliated Hospital of Qingdao University and 58 HCC tissues from the Institute of Biomedical Sciences, Tokushima University. First-strand complementary DNA (cDNA) was synthesized by using the Superscript II Reverse Transcriptase Kit (Invitrogen, Carlsbad, CA, USA).

### Cell transfection and lentiviral transduction

Oligonucleotide TTYH3 siRNA and plasmid were purchased from Jima (Shanghai, China) and Jikai (Shanghai, China), respectively. The sequences of two TTYH3 small interfering RNAs (siRNAs) are shown in Table S4. The siRNA was transfected into cells with Xfect^TM^ RNA Transfection Reagent (Takara, Kyoto, Japan), and the efficiency of siTTYH3#1 and siTTYH3#2 was shown in the Figure S1A. Lipofectamine 2000 (Invitrogen) was used to transfect the plasmid. TTYH3 overexpression lentivirus and the control lentivirus were purchased from Hanbio (Shanghai, China). HCCLM3 and Hep3B cells were infected with concentrated virus and cultured with complete culture media after 24 h. The cells were then selected by puromycin to generate a stable cell line.

### Cell viability and colony formation assay

Cell viability was measured with a CCK-8 kit (Dojindo Labs, Kumamoto, Japan). The cells were seeded in 96-well plates and incubated overnight. Then, cells were transfected with the corresponding siRNA or plasmids. After 48 h, 10 μL CCK-8 reagent was added to each well of the plate. The plate was incubated at 37 °C for 1 h and OD values were measured at an absorbance of 450 nm. For the colony formation assay, cells were inoculated into 6-well plates (1,000 cells/well) and cultured for 14 days. Colonies were fixed with paraformaldehyde and then stained with crystal violet. Finally, colony numbers in the 6-well cell culture cluster were counted to determine colony forming ability.

### Wound healing assays

The cells were inoculated in 12-well plates and incubated at 37 °C for 24 h. The apposed cells were scratched with a 200 µL pipette tip. Then, cells were washed with PBS to remove cell debris and incubated in DMEM without fetal bovine serum at 37 °C. Images were taken 24 h later. The wound healing area was measured using ImageJ software (NIH, Maryland, USA) and mobility was calculated.

### Invasion and migration assays

The invasion experiment was performed in a 24-well Millicell chamber. The 8-μm pore inserts were coated with 30 μg Matrigel (BD Biosciences, New Jersey, USA). Cells (1.0 × 10^5^) in 200 μL serum-free medium were added to the upper chambers and 500 μL DMEM containing 20% fetal bovine serum was added to lower chambers as a chemoattractant. After 24 h incubation, the invading cells were fixed with methanol and stained with 0.5% crystal violet. The staining components in the upper chamber were removed, and then the cells were photographed. Migrated cells were quantified in three random fields. The migration experiment was carried out in a similar manner without Matrigel coating.

### Actin tracker red rhodamine immunofluorescence

Actin tracker red rhodamine immunofluorescence staining was used to analyze the cell skeleton and microfilaments. Cells grown on cover slides and then incubated with phalloidin-rhodamine and DAPI dihydrochloride (Beyotime Institue of Biotechnology, Jiangsu, China). Actin tracker red rhodamine microfilaments were observed and analyzed with fluorescence microscope.

### Apoptosis analysis

The flow cytometry apoptosis assay was performed by a fluorescence microscope (Olympus IX50; Olympus Corp., Tokyo, Japan), and the HCC cells were treated with AlexaFluor® 488 Annexin V/Dead Cell Apoptosis Kit (Invitrogen).

### Histological and immunohistochemical analysis

The 49 HCC tissues from the Affiliated Hospital of Qingdao University including tumor and nontumor samples were fixed with paraformaldehyde, embedded in paraffin and sectioned, stained with hematoxylin and eosin (H&E), and observed via light microscopy. To assess TTYH3 protein expression in clinical specimens and Ki-67 protein expression in mouse tumor tissues, TTYH3 antibody and Ki-67 antibody were incubated with tissue sections at 4 °C overnight. Immunohistochemistry with the streptavidin peroxidase-conjugated method was used for detection.

### Xenograft tumor formation model

Stable HCCLM3 cells (1×10^6^) were injected subcutaneously into the backs of 4-week-old male BALB/c nude mice (Beijing Vital Laboratory Animal Technology Co., Ltd., Beijing, China; n = 7 per group) to induce tumor formation. Over the next four weeks, the animals were raised, and the tumor volume was measured every three days using the following equation: volume (mm^3^) = length × width^2^ × 0.5. Mice were sacrificed at day 27 after injection and the tumor tissue was embedded in paraffin. HE staining was used for pathological observation. In addition, Ki-67 protein expression was analyzed by immunohistochemical experiments. All animal experiments were performed in the Animal Center of Qingdao University in accordance with the procedures approved by the Medical Experimental Animal Care Commission of Qingdao University.

### Lung metastasis model

BALB/c nude mice were injected with 2 × 10^6^ stable HCCLM3 cells in 100 μL PBS via tail vein. The animals were sacrificed after four weeks, and the number of nodules in the lungs was counted. Thereafter, the lung tissue was embedded in paraffin, and pathology of tumor nodules was examined by H&E staining.

### Calcium influx detection

Cells were incubated with Fluo-3 AM (Solarbio, Beijing, China) calcium fluorescence probe in D-Hank's balanced salt solution. After washing, the fluorescence intensity was quantified by fluorescence microscopy and flow cytometry at a wavelength of 490 nm.

### Intracellular chloride ion level assay

Intracellular chloride ion concentrations were determined using N-[ethoxycarbonylmethyl]-6-methoxyquinoline bromide (MQAE) (Beyotime Biotechnology, Shanghai, China), a fluorescent chloride ion indicator that quenches MQAE upon binding to halide ions such as chloride. The binding results in a decrease in fluorescence without a change in wavelength. HCCLM3 and Hep3B cells were incubated with Krebs-HEPES buffer containing 5 mM MQAE for 50 min at 37 °C in a 5% CO^2^ incubator, and then washed five times with Krebs-HEPES buffer. Cells loaded with MQAE were then placed on slides with glycerol for 20 min at room temperature of 24 to 26 °C. Imaging was performed using a Leica microscope to collect and quantify the chlorine-dependent fluorescence signal.

### Western blotting

Total proteins extracted directly from cell lysates with RIPA buffer (Beyotime Biotechnology, Shanghai, China) were quantified with Enhanced BCA Protein Assay Kit (Beyotime Biotechnology, Shanghai, China). Equal amounts of total protein (30 μg) were separated via SDS-PAGE (Beyotime Biotechnology, Shanghai, China) at 100 V for 1.5 h and then transferred to polyvinylidene difluoride membranes (Merck Millipore, Massachusetts, USA). After blocking with 5% skim milk powder for 1.5 h at room temperature, the membranes were incubated overnight at 4 °C with primary antibody, followed by HRP-conjugated secondary antibody (1:10,000) for 1.5 h at room temperature. After washing three times in PBST, the bands were visualized using ECL Western Blotting Substrate (KF005, Affinity, Massachusetts, USA). The band intensity was analyzed with ImageJ. The protein band intensity of GAPDH was set to the control value of 1, and then the relative value of the gray level of other band intensity was calculated.

### Immunoprecipitation (IP) and immunoblotting

HCCLM3 or Hep3B medium was removed and samples were washed with cold PBS. Appropriate amounts of protease inhibitor mixture were then added to prepare lysis buffer (reagents for Capturem IP and CO-IP kits; Takara). The lysates were incubated on ice for 15 min, after which the lysates were collected and centrifuged at 4 °C and 1,000 × *g* for 10 min. After centrifugation, the supernatant was removed. After incubation with the recommended amount of antibody and clarifying the lysate for 20 minutes at room temperature, IP analysis was performed using the Capturem IP and CO-IP kits (Takara) according to the manufacturer's instructions. The collected samples were then analyzed using SDS-PAGE and western blotting analysis, as described above.

### Calcium concentration detection in tissue

Calcium ions were combined with methylthymol blue in alkaline solution to form a blue complex. The calcium content in the tissue can be calculated by the colorimetric method via comparison with the calcium standard of the same treatment. The calcium concentration in HCC tissues was analyzed using a calcium concentration kit (Nanjing Jiancheng Bioengineering Institute, Nanjing, China, C004-2-1) according to the manufacturer's instructions.

### Data mining

The Oncomine (https://www.oncomine.org) and Ualcan databases (http://ualcan.path.uab.edu/index.html) 23 were used to investigate TTYH3 expression across studies involving HCC. The GEPIA database (http://gepia.cancer-pku.cn/index.html) 24 was used to examine the correlation of TTYH3 overexpression with patient survival. The DNA methylation level of TTYH3 in HCC was also analyzed in the Ualcan database. The Kaplan-Meier Plotter database (http://kmplot.com/) 25 was used to explore the correlation of patient survival with TTYH3 and MK5 overexpression. The chipBase database (http://rna.sysu.edu.cn/chipbase/) 26 was used to analyze the correlation between TTYH3 and MK5 expression. The cBioPortal for Cancer Genomics (www.cbioportal.org) 28, 29 database and IntOGen-Cancer Mutations Browser (www.intogen.org) 30 were used to explored TTYH3 mutation.

### Illumina Infinium HumanMethylation850K bead chip and data analysis

HCC tumor and non-tumor tissues from 7 patients were performed Genome-wide DNA methylation analysis. DNA was isolated from whole blood in all studies using a DNeasy Blood and Tissue Kit (Qiagen, Dusseldorf, Germany). The purity and concentration of DNA was estimated using Nanodrop 2000 (Thermo Scientific, Massachusetts, USA). Approximately 500 ng genomic DNA from each sample was used for sodium bisulfite conversion using the EZ DNA methylation Gold Kit (Zymo Research, Los Angeles, USA) following the manufacturer's standard protocol. Genome-wide DNA methylation was assessed using the Illumina Infinium HumanMethylation850K BeadChip (Illumina Inc, California, USA) according to the manufacturer's instructions. The array data (.IDAT files) were analyzed using ChAMP package in R for deriving the methylation level. The methylation status of all the probes was denoted as β value, which is the ratio of the methylated probe intensity to the overall probe intensity (sum of methylated and unmethylated probe intensities plus constant α, where α = 100). CpG sites with |Δβ| ≥0.20 (in test vs. control) and adjusted P-value ≤0.05 were considered differentially methylated sites. A CpG was considered hypermethylated if Δβ ≥ 0.20 or hypomethylated if Δβ ≤ -0.20. The average β values of promoters and CpG islands (CGIs) were compared between disease and normal tissues. Promoters and CGIs with |Δβ| ≥0.20 and adjusted P ≤0.05 were considered for further analysis.

### Pyrophosphate sequencing analysis

A total of 23 paired HCC tumor and non-tumor tissue were performed Pyrophosphate sequencing analysis. DNA extraction was conducted using a genomic DNA extraction kit (Qiagen DNeasy kit, catalog number 69506). DNA Methylation treatment was conducted using the QiagenEpiTect Bisulfite Kit (Qiagen, #59104). The primer was designed with PyroMark Assay Design 2.0. Primer sequence information is displayed in Table S4. Then, RNA was amplified by PCR and pyrosequencing was performed (PyroMark Q96 ID, Qiagen).

### Protein docking

The global docking of TTYH3 and MK5 was performed by ClusPro (http://nrc.bu.edu/cluster) 30. The conformation with the highest comprehensive score was visualized by PyMol (The PyMOL Molecular Graphics System, Version 2.0 Schrödinger, LLC.) and the binding sites of the two proteins were obtained. Subsequently, the TTYH3 residues involved in the formation of the two proteins were analysis by alanine scan. Site directed mutagenesis were constructed by PyMOL.

### Statistical analysis

Results were presented as the mean ± SEM of three or more repeated experiments. The comparison of means between two groups was conducted by using Student's *t*-test. P <0.05 was considered statistically significant. The Cox proportional hazards models were used to identify independently significant variables. The Kaplan-Meier method was used to compare survival by different levels of mRNA expression. Spearman's correlation coefficient was calculated using the gene copy number and its corresponding mRNA expression. Correlations were considered significant and positive when P < 0.05 and r > 0.30.

## Results

### TTYH3 promotes cellular proliferation, migration, and invasion and inhibits HCC cell apoptosis *in vitro*

**Figure 1:**
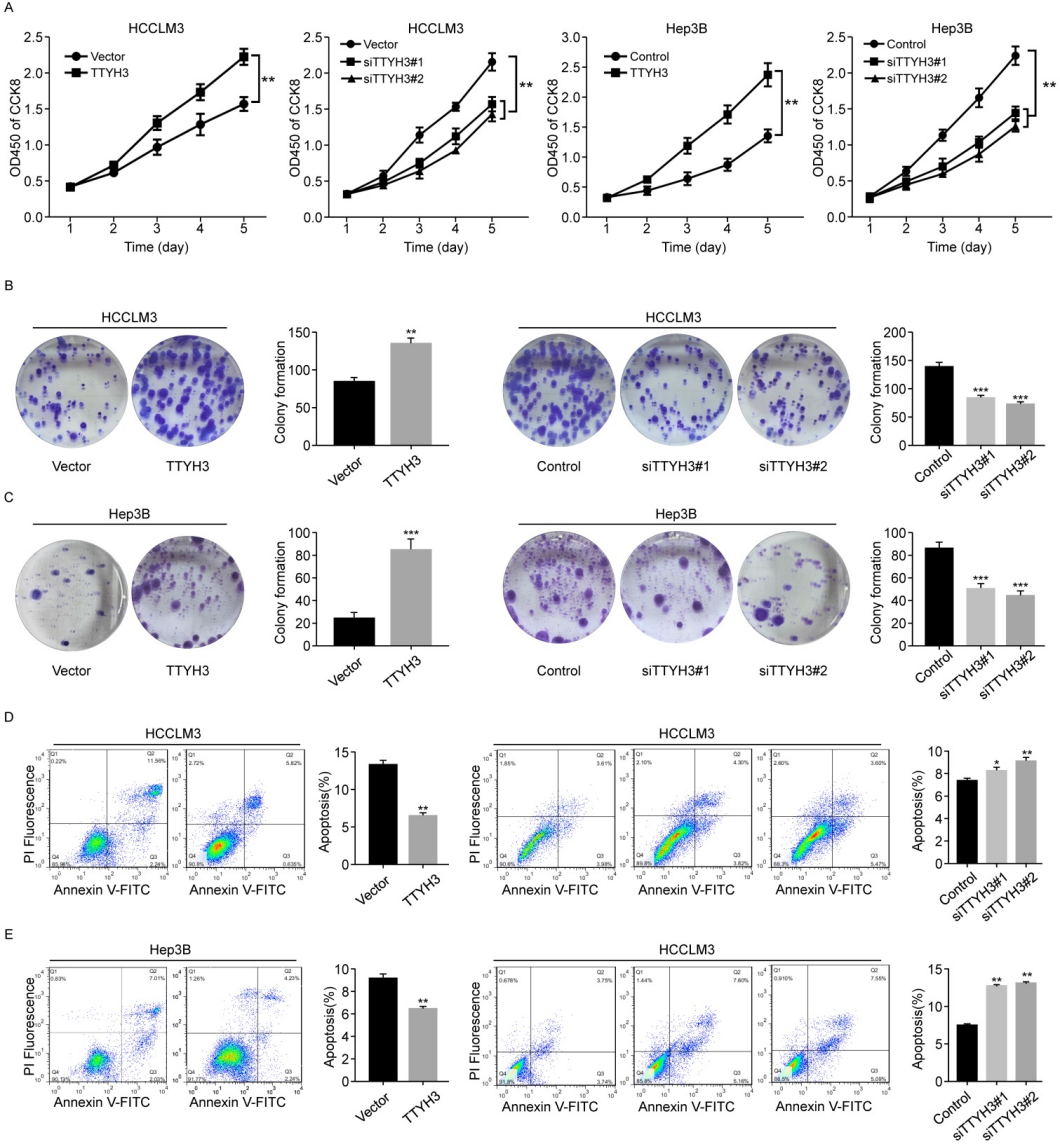
** TTYH3 promoted HCC cell proliferation and inhibited HCC cell apoptosis. (A, B, C)** The proliferation of HCCLM3 and Hep3B cells transfected with vector, TTYH3, control, siTTYH3#1, and siTTYH3#2 were detected by CCK-8 and colony formation assays. **(D, E)** The apoptosis of HCCLM3 and Hep3B cells transfected with vector, TTYH3, control, siTTYH3#1, and siTTYH3#2 were detected by flow cytometry. Statistically significant differences are as follows: *P < 0.05; **P < 0,01; ***P < 0.001; Student's *t*-test. The experiment was repeated at least three times.

**Figure 2:**
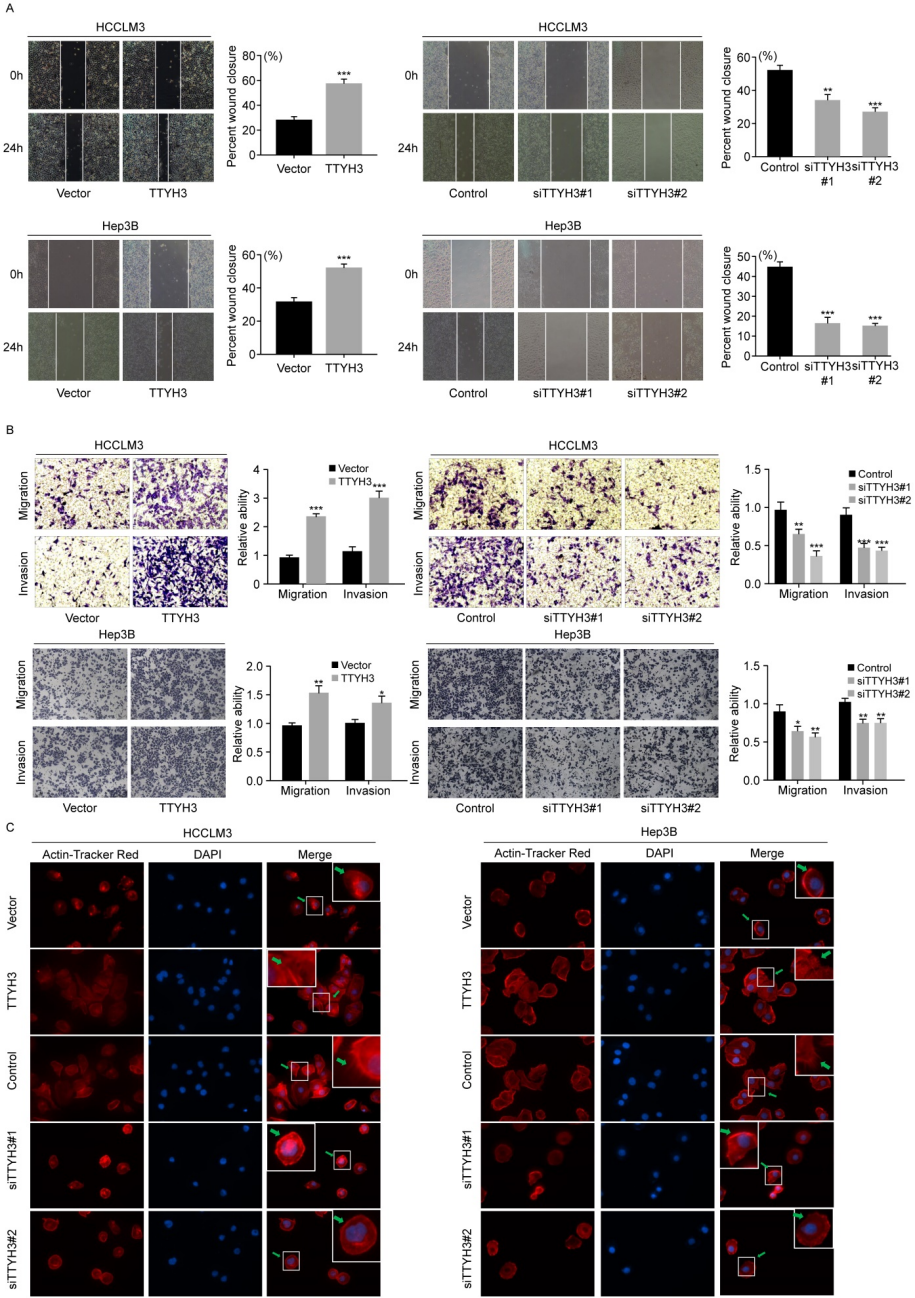
** TTYH3 promoted the cellular motility of HCC cells. (A, B)** The migration of HCCLM3 and Hep3B cells transfected with vector, TTYH3, control, siTTYH3#1, and siTTYH3#2 were detected by wound healing and Transwell assays. Statistically significant differences are as follows: *P < 0.05; **P < 0,01; ***P < 0.001; Student's *t*-test. The experiment was repeated at least three times. **(C)** Actin tracker red rhodamine immunofluorescence staining was used to analyze the cell skeleton, microfilaments and movement ability.

We first investigated the expression of TTYH3 in 7 common HCC cell lines. HCCLM3 and Hep3B cell lines were selected for further study as the TTYH3 expression was highest in HCCLM3 and lowest in Hep3B, respectively (Figure S1B). According to CCK-8 assays and cloning formation, TTYH3-overexpressing (TTYH3-OE) cells showed significantly higher cell proliferation (Figure 1A) and more colony formation (Figure 1B and 1C) compared to control vector. In contrast, TTYH3 knockdown by siRNA inhibited cell proliferation and colony formation (Figure 1A-C). Flow cytometry indicated that TTYH3 overexpression significantly decreased the proportion of cellular apoptosis, and TTYH3 knockdown showed the opposite effect (Figure 1D and 1E). To evaluate the effect of TTYH3 on cell motility, we performed wound healing and Transwell assays. The wound healing rate was significantly higher in TTYH3-OE cells compared to that in control cells, and the opposite result was observed in TTYH3 knockdown cells (Figure 2A). Transwell assays showed that TTYH3 overexpression significantly promoted cell migration and invasion, while TTYH3 knockdown attenuated cell migration (Figure 2B). Actin tracker red rhodamine immunofluorescence staining was used to analyze the cell skeleton and microfilaments (Figure 2C). The results showed that the cell skeleton and microfilaments in TTYH3 overexpression cells were more than control group, which suggested that the movement ability of TTYH3 overexpression cells increases. While TTYH3 knockdown reduced cell skeleton, microfilaments and movement ability.

### TTYH3 promotes tumor formation and HCC cell metastasis *in vivo*

**Figure 3:**
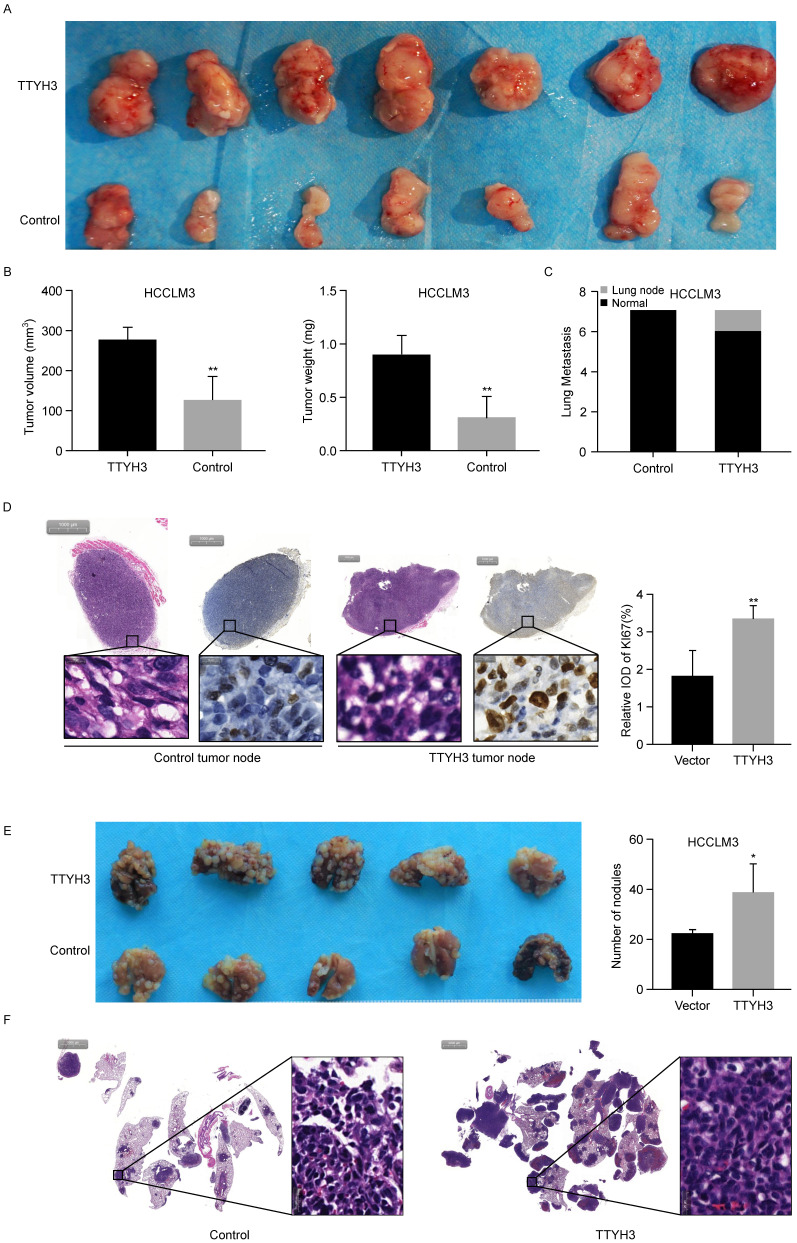
** TTYH3 promoted tumor formation in the subcutaneous xenograft model (n = 7), HCCLM3 cell metastasis in the metastasis model (n = 5). (A)** Stable TTYH3 or control HCCLM3 cells were injected subcutaneously in seven nude mice. Tumors were collected after four weeks. **(B)** Comparison of tumor volume and weight between the control group and the group injected with HCCLM3 cells stably expressing TTYH3. **(C)** Lung metastasis occurred in one mouse in cells stably expressing TTYH3. **(D)** Hematoxylin and eosin (HE) staining and immunohistochemical staining of Ki-67 in the tumor node of the control group and cells stably expressing TTYH3. **(E)** TTYH3-overexpressing (TTYH3-OE) or control stable HCCLM3 cells were injected into the tail veins of nude mice. After four weeks, the lung tissues were collected. Comparison of the number of tumor nodules in the control and TTYH3-OE stable HCCLM3 cells. **(F)** Hematoxylin and eosin (HE) staining of lung tissues showing the tumor nodules. Statistically significant differences are as follows: *P < 0.05; **P < 0,01; ***P < 0.001; Student's *t*-test.

To further investigate the roles of TTYH3 *in vivo*, TTYH3-OE or control stable HCCLM3 cells were injected subcutaneously into nude mice. After four weeks, the formed tumors were collected and the tumor volume and weight were measured (Figure 3A and 3B). The tumor volume and weight were higher in the TTYH3-OE group than in the control group (n = 7, P < 0.01). Furthermore, Ki-67 expression was considerably higher in the tumors in the TTYH3-OE group compared to that in the control group (Figure 3D). Interestingly, lung metastasis occurred in one of the seven mice in the TTYH3-OE group (Figure 3C and Figure S1C). We then injected TTYH3-OE or control stable HCCLM3 cells via the tail vein to investigate the effect of TTYH3 on promoting metastasis. After four weeks, the lung tissues were collected for further study. Metastases were observed in all ten mice injected with both control and TTYH3-OE cells, while there were significantly more metastatic nodules in the TTYH3-OE group (Figure 3E). H&E staining of the lung nodule confirmed the cellular morphology of tumor cells (Figure 3F).

### TTYH3 promotes calcium and chloride influx in HCC cells

**Figure 4:**
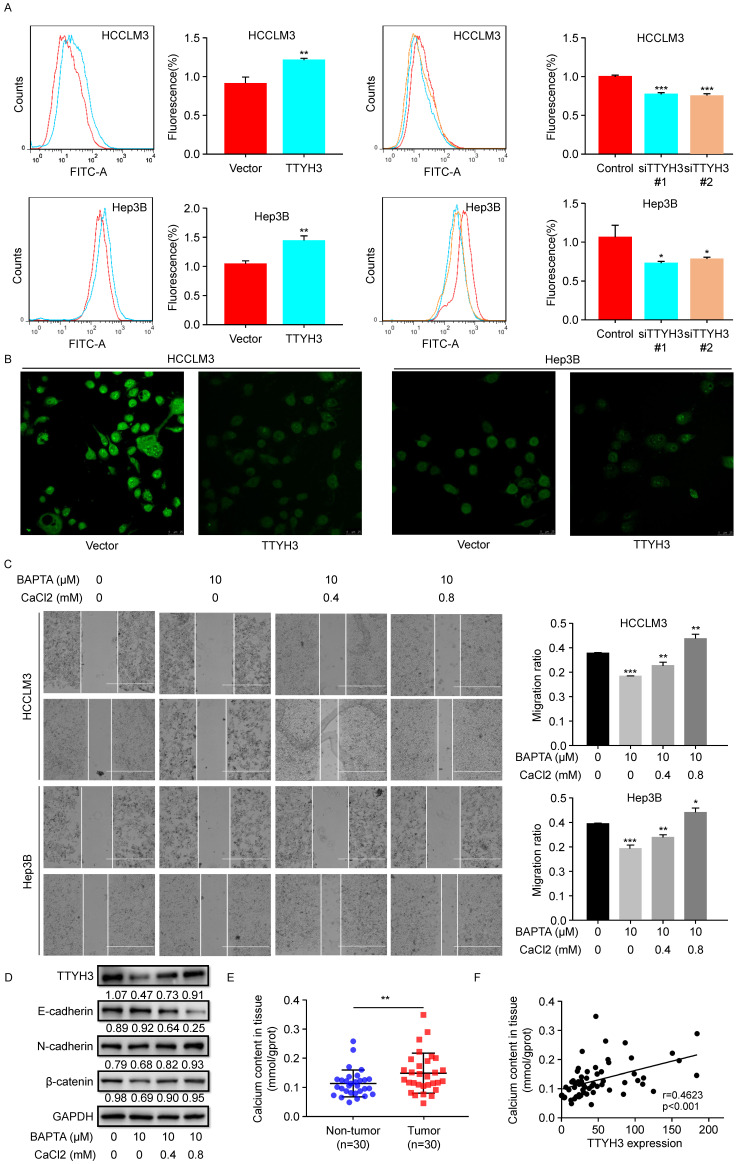
** TTYH3 promoted calcium and chloride influx and epithelial-mesenchymal transition (EMT) in HCC cells. (A)** Calcium influx was detected by flow cytometry after infection of control vector, TTYH3, control siRNA, siTTYH3#1, or siTTYH3#2 in HCCLM3 and Hep3B cells. **(B)** Intracellular chloride concentration was detected by fluorescence after infection of control vector, TTYH3 overexpression in HCCLM3 and Hep3B cells. **(C)** CaCl_2_ promoted the motility of HCC cells by wound healing assays, and motility could be attenuated by the calcium chelator BAPTA. **(D)** CaCl_2_ promoted of TTYH3, E-cadherin, N-cadherin, and β-catenin protein expression, which were attenuated by BAPTA. **(E)** Ca^2+^ concentration in fresh HCC tissue (n = 30). **(F)** The correlation of Ca^2+^ concentration with TTYH3 expression was measured in HCC tissues (n = 30). Statistically significant differences are as follows: *P < 0.05; **P < 0,01; ***P < 0.001; Student's *t*-test.

As TTYH3 is involved in the Ca^2+^-activated chloride channel, we investigated whether Ca^2+^ or Cl^-^ was involved in promoting cancer progression. As indicated by flow cytometry, calcium influx increased when TTYH3 was overexpressed (Figure 4A). Meanwhile, calcium influx decreased when TTYH3 expression was knocked down (Figure 4A). Intracellular chloride ion concentration was measured with N-[ethoxycar bonylmethyl]-6-methoxy-qu inolinium bromide (MQAE), a fluorescent chloride ion indicator. Upon binding chloride, MQAE is quenched, resulting in a decrease in fluorescence without a shift in wavelength. The fluorescence intensity in TTYH3-OE group was lower than that in the control group, indicating increased chloride concentration (Figure 4B and Figure S2A). We then used wound healing assays to demonstrate that CaCl_2_ promoted HCC cell motility, which could be attenuated by the calcium chelator BAPTA (Figure 4C). CaCl_2_ also promoted TTYH3, N-cadherin, and β-catenin protein expression and inhibited E-cadherin expression, and these effects were attenuated by BAPTA (Figure 4D). Then, we measured the Ca^2+^ concentration in fresh HCC tissue. The Ca^2+^ concentration was significantly higher in tumor tissue than in nontumor tissue (Figure 4E, P < 0.01), and Ca^2+^ concentration was correlated with TTYH3 expression (Figure 4F, P < 0.001, r = 0.4623).

### TTYH3 promotes epithelial-mesenchymal transition (EMT) through MK5 in HCC cells

**Figure 5:**
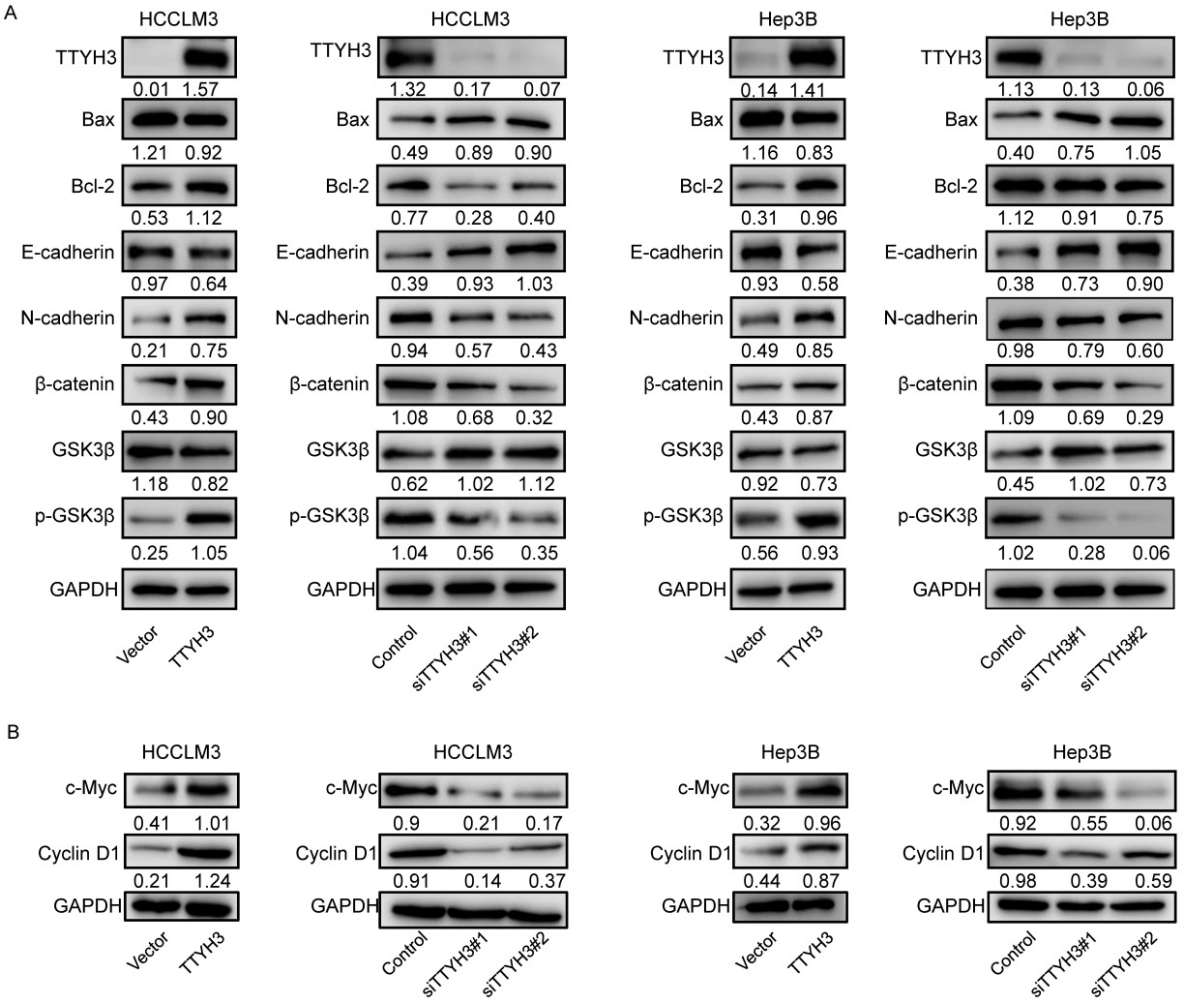
** TTYH3 regulated apoptotic and epithelial-mesenchymal transition (EMT)-related protein expression and GSK3β/β-catenin signaling in HCC cells. (A, B)** HCCLM3 and Hep3B cells were transfected with control vector, TTYH3, control siRNA, siTTYH3#1, or siTTYH3#2. The protein expression Bax, Bcl-2, E-cadherin, N-cadherin, GSK3β, p-GSK3β, β-catenin, c-Myc and Cyclin D1 were investigated by western blot.

To investigate the mechanism by which TTYH3 regulates HCC, we explored whether TTYH3 could regulate the expression of proteins related to apoptosis and EMT. TTYH3 overexpression promoted Bax expression and inhibited Bcl-2 expression, while TTYH3 knockdown had the opposite effect (Figure 5A and 5B). TTYH3 overexpression significantly promoted N-cadherin, β-catenin, c-Myc and Cyclin D1 expression and inhibited E-cadherin expression in HCCLM3 and Hep3B cells (Figure 5A and 5B). The phosphorylation of GSK3β (Ser9) was promoted by TTYH3 overexpression and decreased by TTYH3 knockdown (Figure 5A).

**Figure 6:**
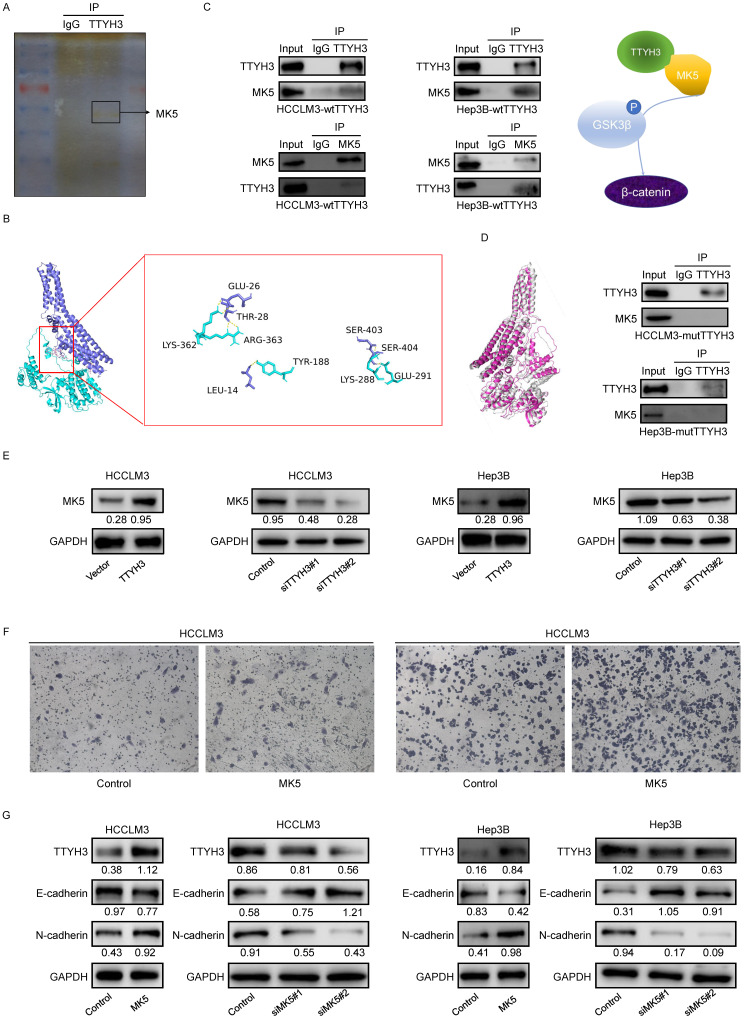
** TTYH3 promotes epithelial-mesenchymal transition (EMT) through MK5 in HCC cells. (A)** The immunoprecipitated proteins were visualized by silver staining and mass spectrometry. The MK5 protein at 54 kDa may be the protein that interacts with TTYH3. **(B)** Docking model analysis of the interaction between TTYH3 and MK5. TTYH3 protein is blue, MK5 protein is green, and the interaction interface is pink. **(C)** Co-immunoprecipitation of TTYH3 and MK5 in HCCLM3 and Hep3B cells. **(D)** Conformations of wild TTYH3 and mutant TTYH3. Co-immunoprecipitation of mutant TTYH3 and MK5 in HCCLM3 and Hep3B cells. Wild TTYH3 is grey, and mutant TTYH3 is pink. **(E)** MK5 expression after transfection of control vector, TTYH3, control siRNA, siTTYH3#1, or siTTYH3#2 in HCCLM3 and Hep3B cells. **(F)** The effect of MK5 expression on cellular motility was tested by Transwell assay. HCCLM3 and Hep3B cells were transfected with control vector or MK5. **(G)** E-cadherin and N-cadherin expression after transfection of control vector, MK5, control siRNA, siMK5#1, or siMK5#2 in HCCLM3 and Hep3B cells.

We then performed mass spectrometry and CO-IP analysis to investigate the molecular mechanism by which TTYH3 promotes EMT. MK5 was one of the immunoprecipitated proteins selected from mass spectrometry (Figure 6A). *TTYH3* mRNA expression was significantly correlated with *MK5* expression in The Cancer Genome Atlas (TCGA) (Figure S2B). Subsequently, we determined the protein docking structure of TTYH3 in complex with the MK5 coiled-coil fragment (Figure 6B). The binding surface suggested that the subunits of the two proteins form a complex by forming non-covalent bonds. Wild type TTYH3 was co-immunoprecipitated with MK5 in HCCLM3 and Hep3B cells (Figure 6C). A structure-guided interaction analysis showed that a mutation of TTYH3 interface residues (termed mutant TTYH3) strongly diminished its interactions with MK5, while the mutations did not alter protein structure (Figure 6D). Subsequently, we determined that protein CO-IP analysis test was negative when the cells were transfected with mutant TTYH3 plasmid. The results for the mutant suggested mutant TTYH3 may reduce binding to MK protein (Figure 6D). Then, MK5 expression was significantly upregulated after TTYH3 overexpression and downregulated after TTYH3 knockdown (Figure 6E). Transwell assay indicated that the cell migration was promoted by MK5 overexpression (Figure 6F). N-cadherin expression was upregulated and E-cadherin was downregulated by MK5 overexpression, and the opposite effect was observed when MK5 expression was knocked down (Figure 6G). Furthermore, the regulation of TTYH3 or MK5 expression was accompanied by a simultaneous change in TTYH3 expression (Figure 6E and 6G).

### TTYH3 activates GSK3β/β-catenin signaling via MK5

**Figure 7:**
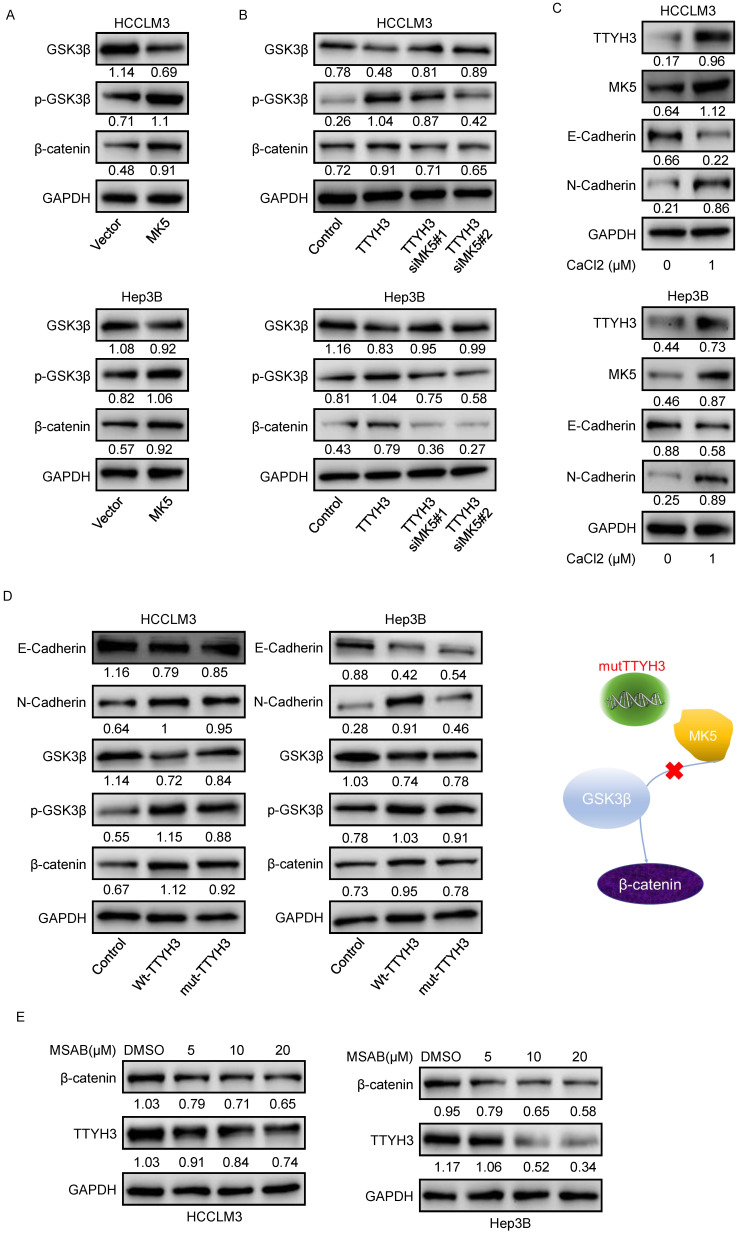
** TTYH3 activates GSK3β/β-catenin signaling by MK5. (A)** GSK3β phosphorylation and protein expression after infection of control vector and MK5. **(B)** GSK3β phosphorylation and protein expression after control vector, TTYH3, TTYH3 with siMK5#1, or TTYH3 with siMK5#2 in HCCLM3 and Hep3B cells. **(C)** TTYH3, MK5, E-cadherin, and N-cadherin expression were analyzed after CaCl_2_ treatment. **(D)** Protein expression of E-cadherin, N-cadherin, GSK3β, p-GSK3β and β-catenin after transfection with wild-type TTYH3 or mutant TTYH3 were measured by western blot. **(E)** TTYH3 protein expression after treatment of MSAB, a β-catenin inhibitor, was measured by western blot.

As GSK3β is one of the upstream proteins of β-catenin, we investigated whether TTYH3 and MK5 activate GSK3β. The phosphorylation of GSK3β (Ser9) and β-catenin levels were upregulated by MK5 overexpression and downregulated by MK5 knockdown (Figure 7A). In the rescue study, the levels of p-GSK3β (Ser9) and β-catenin expression were attenuated by MK5 knockdown in TTYH3-OE cells (Figure 7B). We then examined whether CaCl_2_ was involved in the regulation of MK5 and EMT-related protein expression in HCC cells. TTYH3, MK5, and N-cadherin expression were upregulated and E-cadherin was downregulated by treating cells with CaCl_2_ (Figure 7C). And the mutant TTYH3 attenuated the expression of p-GSK3β (Ser9), β-catenin and N-cadherin compared to wild-type TTYH3 transfection (Figure 7D). The results suggested that *TTYH3* mutation may weaken the binding of TTYH3 to MK protein. The signaling activation was attenuated (Figure 7D). Moreover, the treatment of MASB, a β-catenin inhibitor, inhibited the TTYH3 expression in a dose dependent manner (Figure 7E).

### TTYH3 is overexpressed in HCC tissue and correlates with poor patient survival

**Figure 8:**
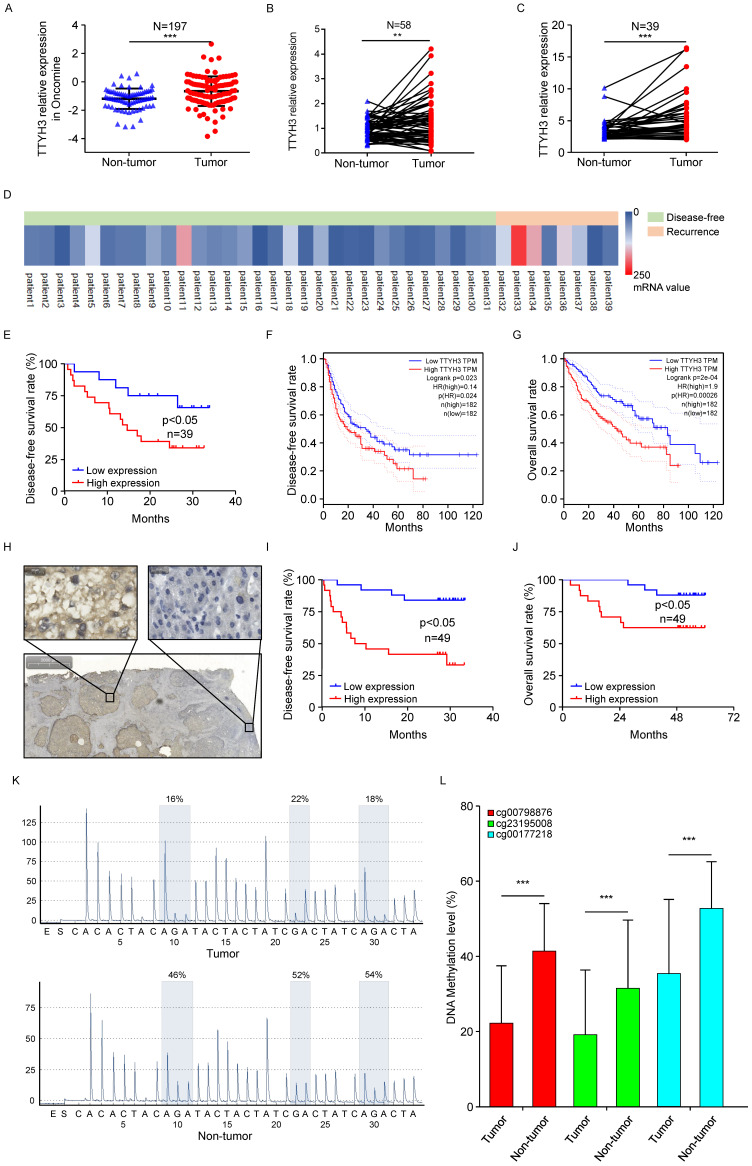
** TTYH3 was highly expressed in HCC tumor tissue and associated with patient disease-free survival (DFS) and overall survival (OS). (A)** Comparation of relative TTYH3 expression in nontumor and tumor tissues in the Oncomine database (A). **(B, C)** Comparation of relative TTYH3 expression in nontumor and tumor tissues in our two patient cohorts. **(D)** The correlation of TTYH3 expression with tumor recurrence in an RNA sequencing dataset. **(E)** High TTYH3 expression was associated with poor DFS in our patient cohort. **(F, G)** High TTYH3 expression was associated with poor DFS and OS in TCGA database. **(H)** Immunohistochemistry (IHC) of TTYH3 in HCC. **(I, J)** High TTYH3 protein expression was associated with poor DFS and OS in our patient cohort. **(K, L)** Pyrophosphate sequencing analysis confirmed that the methylation level of three CpG sites in HCC tissues was lower in tumor tissue than in nontumor tissue. Statistically significant differences are as follows: *P < 0.05; **P < 0,01; ***P < 0.001; Student's *t*-test; Kaplan-Meier analysis.

According to the Oncomine dataset (Figure 8A), *TTYH3* expression was higher in tumor tissue compared to that in nontumor tissue. We then validated gene expression in our patient cohort from the Institute of Biomedical Sciences, Tokushima University and the Affiliated Hospital of Qingdao University. *TTYH3* mRNA expression was significantly higher in HCC tissue compared with that in nontumor tissue (Figure 8B and 8C, P < 0.01). Furthermore, high TTYH3 expression was significantly correlated with patient DFS (Figure 8D and 8E). According to TCGA, high *TTYH3* mRNA expression was significantly correlated with poor DFS and OS (Figure 8F and 8G). Immunostaining showed that TTYH3 protein was highly expressed in the tumor cell membrane (Figure 8H), and high TTYH3 protein expression was significantly correlated with poor DFS and OS in patient cohort from the Affiliated Hospital of Qingdao University (Figure 8I and 8J). Univariate and multivariate analyses revealed that TTYH3 was an independent prognostic factor (Table S1, S2 and S3). Bioinformatics analysis also indicated that high TTYH3 or MK5 expression was significantly correlated with disease-free survival (DFS) and OS (P < 0.05, Figure S3 and S4).

To investigate the mechanism of high TTYH3 expression in tumor tissue, we performed DNA methylation microarray with 850K BeadChip in seven paired HCC tissues (Figure S5A). Then, pyrophosphate sequencing analysis confirmed that the methylation level of three CpG sites (CG00798876, CG23195008, and CG00177218) in HCC tissues was lower in tumor tissue than in nontumor tissue (Figure 8K and 8L, n = 23, P < 0.05). Bioinformatics analysis in TCGA database also confirmed that there was DNA hypomethylation in the tumor tissue (Figure S5B). The cBioPortal database analysis (Figure S5C) indicated that only one HCC case had TTYH3 gene amplification out of 372 cases from TCGA database. We further explored TTYH3 mutation through the IntOGen-Cancer Mutations Browser database, and the result also shown the mutation rate of TTYH3 is very low, and the mutations needle plot shown the distribution of the observed mutations along the protein sequence (Figure S5D).

## Discussion

Ion channels participate in various pathophysiological functions of cancer progression 4. Here, we found that the large conductance chloride channel TTYH3 promoted cell proliferation, migration, and invasion and inhibited cellular apoptosis in HCC cells. Furthermore, TTYH3 promoted tumor formation and metastasis in a mouse model. The transportation of calcium and chloride by TTYH3 may stimulate EMT by promoting MK5 expression and activating the GSK3β/β-catenin signaling pathway. In terms of its clinical significance, high TTYH3 expression in HCC tissue was correlated with poor patient DFS and OS. The promoter and CpG sites in the promoter were hypomethylated in tumor tissue.

Human TTYHs, which are homologs of *tweety* in flightless Drosophila, are widely expressed in the embryo stage and central nervous system (CNS) tissues. They are also expressed in a wide range of mature organisms. In vertebrates, TTYH1 is expressed mainly in the CNS, while TTYH2 is expressed mainly in the liver and adrenal gland. TTYH3 is mainly expressed in the spleen, lungs, and immune cells 14, 31, 32. Among the TTYHs, TTYH3 is commonly upregulated in cancer, including esophageal carcinoma, liver cancer, lung cancer, pancreatic cancer, as shown by microarray and RNA-seq analyses 31, 33. According to gene expression assay, TTYH3 is upregulated in brain and colon cancer 33. In the present study, we found that TTYH3 was highly expressed in HCC tissues, and TTYH3 upregulation was significantly correlated with patient DFS and OS. The common overexpression of TTYH3 in cancers and its correlation with patient survival in HCC indicates that it may serve as a promising biomarker for predicting patient prognosis.

TTYHs are involved in various biological functions, including embryonic development 13, 14, brain physiology and pathology 15, and tumor progression 16, 17. TTYH1 is strongly associated with brain cancer, while TTYH2 promotes colon cancer and osteosarcoma progression 17, 34. However, compared to TTYH1 and TTYH2, TTYH3 has not been studied as extensively. TTYH3 may regulate immune cell activation in response to pathogen-associated molecular patterns 25 and may be involved in neural development, as it is highly expressed in neural tissue 31. TTYH3 is also upregulated in gastric cancer and was found to be correlated with patient prognosis via bioinformatics analysis 22. In the present study, we showed that TTYH3 promoted cellular proliferation, invasion, and migration and inhibited cellular apoptosis of HCC cells. Furthermore, TTYH3 was found to induce resistance to lenvatinib, a drug used for targeted therapy of HCC (Figure S6). In addition to its individual functions, TTYH3 may also promote tumorigenesis by fusion with BRAF serine/threonine kinase (TTYH3-BRAF fusion protein) in glioblastoma, histiocytic sarcoma, and thyroid carcinoma 36-38.

Chloride channels are involved in different cellular and physiological processes. Although TTYH3 has been identified as a gated chloride channel, its biochemical and subsequent biological functions have not been widely studied, especially in cancers. As a large conductance chloride channel, TTYH3 activation is calcium-dependent, swelling-dependent, and volume-regulated 12, 21, 39. The activity of the TTYH3 channel has been found to depend on the intracellular Ca^2+^ concentration 21. In the present study, we showed that Ca^2+^ influx increased when TTYH3 expression was upregulated and decreased when TTYH3 expression was knocked down. Furthermore, Cl^-^ uptake was also promoted by TTYH3 overexpression, as shown by immunofluorescence. After treatment with CaCl_2_, the cellular migration ability was increased and could be blocked by BAPTA. In HCC tissue, the Ca^2+^ content was much higher than that in nontumor tissue, and the Ca^2+^ content was significantly correlated with *TTYH3* mRNA expression. The results indicated that TTYH3 may promote cancer progression by transporting calcium and chloride into cancer cells. CaCl_2_ also promoted TTYH3 expression, demonstrating the positive feedback of TTYH3 expression in HCC.

Calcium-activated chloride channels are a group of chloride channels including the CLCA family, bestrophin family, tweety family, and TMEM16A 40, 41. Members of the CLCA family, including CLCA1, CLCA2, and CLCA4, inhibit tumor proliferation and metastasis in various cancers 11, 42, 43. TMEM16A is the most well-studied Ca^2+^-activated chloride channel in cancers 41. TMEM16A activates different signaling pathways in different cancers, such as the EGFR and CAMKII signaling pathway in breast cancer, the p38 and ERK1/2 signaling pathway in hepatoma, the Ras-Raf-MEK-ERK1/2 signaling pathway in head and neck squamous cell carcinoma and bladder cancer, and the NF-κB signaling pathway in glioma 41. In our study, TTYH3 showed significant effects on HCC cell invasion and migration. Considering that EMT is important during tumor progression, we investigated EMT-related protein expression. We found that E-cadherin decreased and N-cadherin increased after TTYH3 overexpression and CaCl_2_ treatment. The Wnt/β-catenin signaling pathway is involved in regulating EMT, and our results showed that p-GSK3β, β-catenin, c-Myc and Cyclin D1 expression was upregulated by TTYH3. Furthermore, results of mass spectrometry and CO-IP indicated that MK5 was precipitated with TTYH3. The docking model indicated that TTYH3 combined with MK5 via hydrogen bonds between amino acid residues. MK5 expression was consistent with TTYH3 and CaCl_2_ treatment, and MK5 overexpression increased the level of GSK3β and β-catenin phosphorylation. MK5 knockdown attenuated the p-GSK3β and β-catenin expression induced by TTYH3. Thus, we showed that TTYH3 may activate GSK3β/β-catenin signaling by promoting MK5 expression.

**Figure 9:**
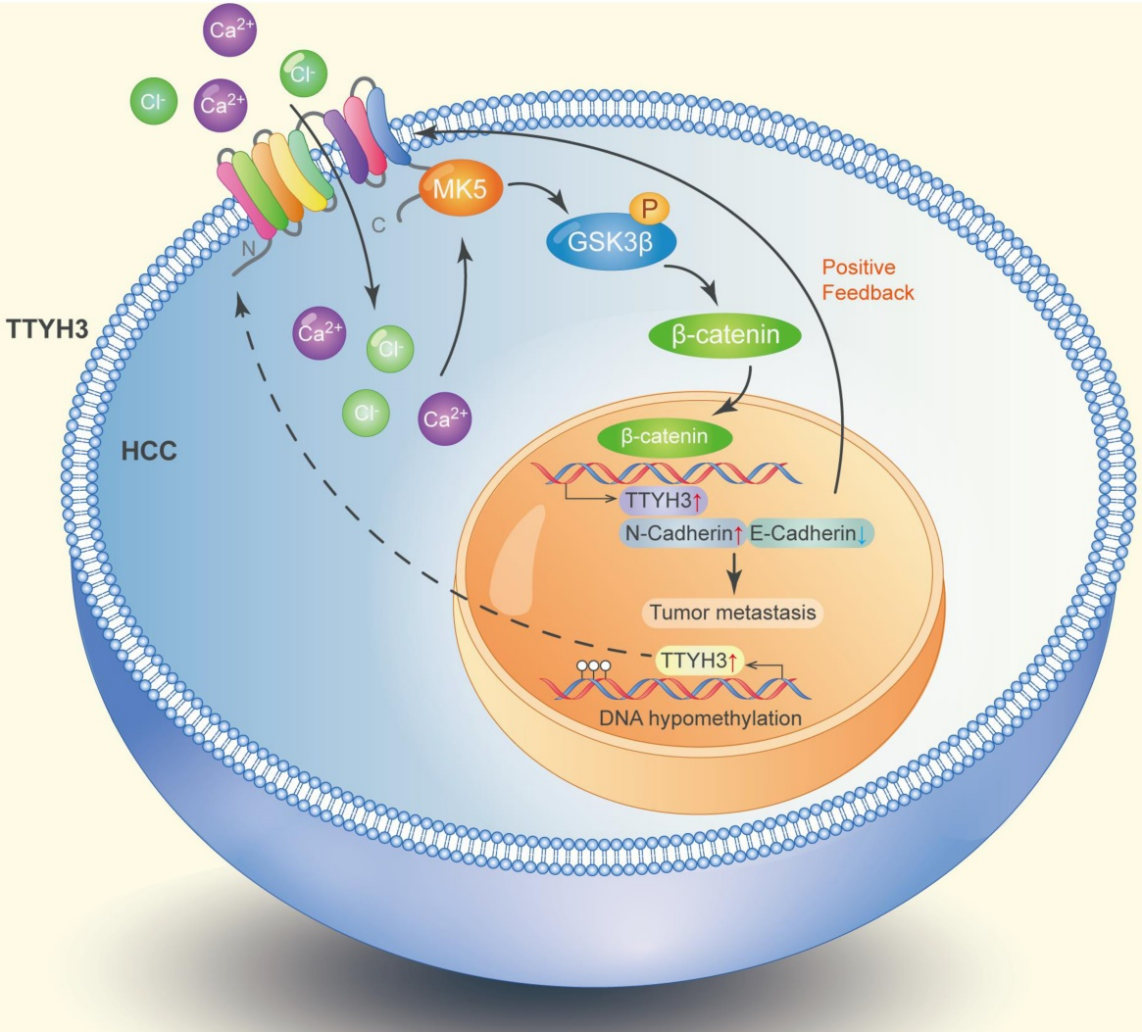
Diagram of the molecular function of TTYH3 in HCC metastasis.

TTYH3 was highly expressed in HCC tissue and correlated with poor patient survival. Gene overexpression can be caused by gene amplification, epigenetic regulation, transcriptional regulation, or post-translational regulation. The cBioPortal database and the IntOGen-Cancer Mutations Browser database shown the mutation rate of TTYH3 is very low. Furthermore, bioinformatics analysis indicated that DNA methylation may regulate TTYH3 expression. In our study, DNA methylation microarray and pyrophosphate sequencing analysis showed that TTYH3 was hypomethylated in the promoter area and CpG islands. DNA hypomethylation in the tumor promoter may be one of the reasons for TTYH3 upregulation in HCC tissue. Moreover, the TTYH3 could regulate its own expression via positive feedback manner, as indicated by the high expression of TTYH3 after treatment with CaCl_2_ or transfection of TTYH3 or MK5 or treat the cell with β-catenin inhibitor. The molecular function of TTYH3 in HCC metastasis was shown in the diagram (Figure 9).

In conclusion, we identified that TTYH3 promotes HCC progression through MK5/GSK3β/β-catenin signaling by transporting Ca^2+^ and Cl^-^ into the cytoplasm. The high expression of TTYH3 may be regulated by DNA methylation and positive feedback manner. Our study provides new insight into the role of chloride channels in regulating tumor metastasis. Furthermore, we suggest that TTYH3 could be a predictive biomarker for HCC prognosis and serve as a target for its treatment.

## Supplementary Material

Supplementary figures and tables.

## Abbreviations

OS: overall survival
RNA-seq: RNA sequencing
H&E: hematoxylin and eosin
IP: immunoprecipitation
EMT: epithelial-mesenchymal transition
