# Supplementary material for: The TTYH3/MK5 Positive Feedback Loop regulates Tumor Progression via GSK3-β/β-catenin signaling in HCC
Source: Int J Biol Sci. 2022 Jun 21;18(10):4053–70. doi: 10.7150/ijbs.73009 (PMC9274494; doi:10.7150/ijbs.73009)
Supplement: Supplementary file 1 — Supplementary figures and tables. [file ijbsv18p4053s1.pdf]

Supplemental Figure S1. (A) The efficiency of siTTYH3#1 and siTTYH3#2. (B) TTYH3 expression in different cell lines. (C) H&E and immunohistochemical staining of Ki-67 in the lung tissues of the control group and cells stably expressing TTYH3.

Supplemental Figure S2. (A) The staining intensity of the fluorescence chloride-probe intensity. (B) The correlation of *TTYH3* mRNA expression with *MK5* was investigated using The Cancer Genome Atlas (TCGA) data.

Supplemental Figure S3. High TTYH3 expression was associated with poor DFS and OS in the TCGA database.

Supplemental Figure S4. High MK5 expression was associated with poor DFS and OS in the TCGA database.

Supplemental Figure S5. The CpG sites in the promoter of TTYH3 were hypomethylated in tumor tissue. (A) DNA methylation microarray was performed with an Illumina Infinium HumanMethylation850K BeadChip. (B) The methylation level of the TTYH3 promoter in liver hepatocellular carcinoma (LIHC) from The Cancer Genome Atlas (TCGA) database. (C) Mutation status of TTYH3 in the cBioPortal database. (D) The distribution of the observed mutations along the protein sequence in the IntOGen - Cancer Mutations Browser database.

Supplemental Figure S6. TTYH3 induce the drug resistance of HCCLM3 cells.

Supplemental Table S1. Univariate analysis of continuous variables.

Supplemental Table S2. Univariate analysis of categorical variables.

Supplemental Table S3. Multivariate regression analysis.

Supplemental Table S4. Detailed material information.

Figure S1

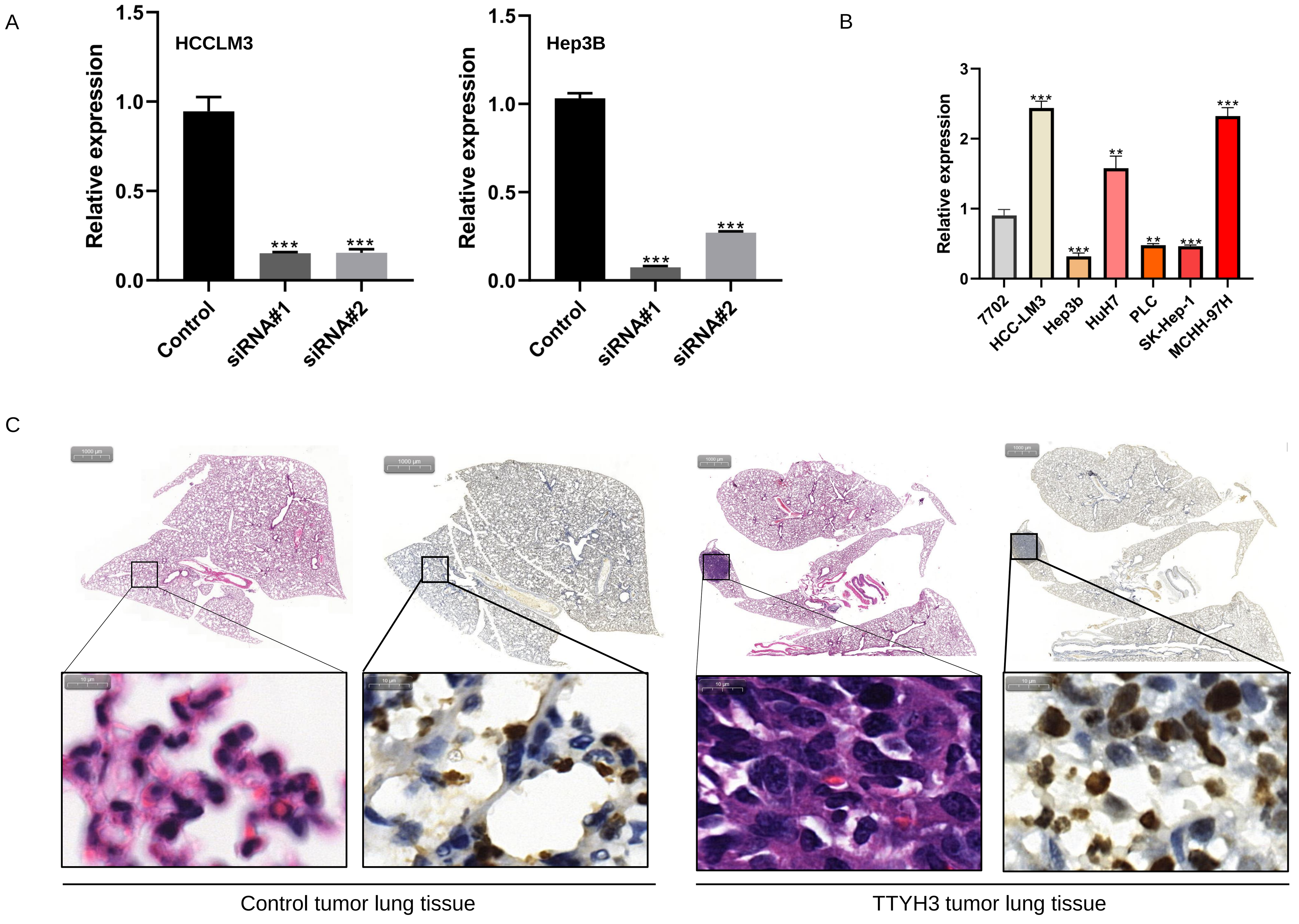
A
HCCLM3
Relative expression
1.5
1.0
0.5
0.0
***
***
Control
siRNA#1
siRNA#2
Hep3B
Relative expression
1.5
1.0
0.5
0.0
***
***
Control
siRNA#1
siRNA#2
B
Relative expression
3
2
1
0
***
***
**
**
***
***
7702
HCC-LM3
Hep3b
HuH7
PLC
SK-Hep-1
MCHH-97H
C
Control tumor lung tissue
TTYH3 tumor lung tissue

Figure S2

A

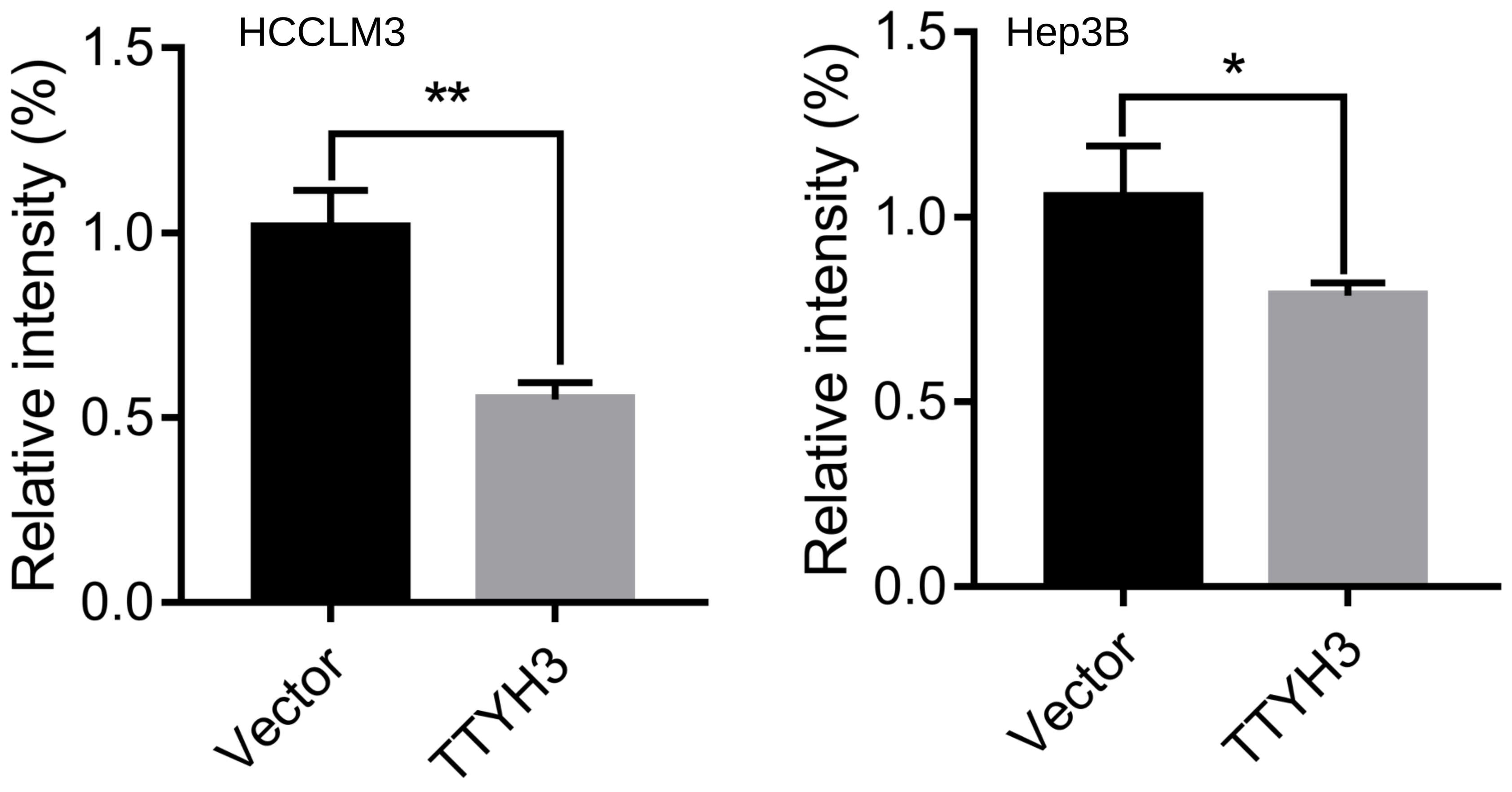
HCCLM3
Relative intensity (%)
1.5
1.0
0.5
0.0
**
Vector
TTYH3
Hep3B
Relative intensity (%)
1.5
1.0
0.5
0.0
*
Vector
TTYH3

B

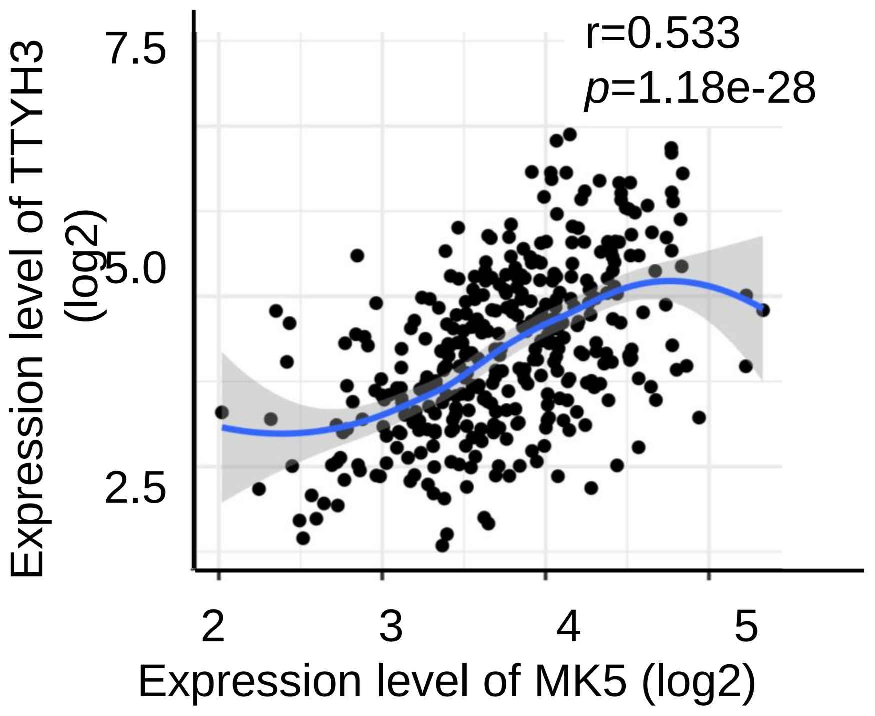
r=0.533
p=1.18e-28
Expression level of TTYH3 (log2)
7.5
5.0
2.5
2
3
4
5
Expression level of MK5 (log2)

Figure S3

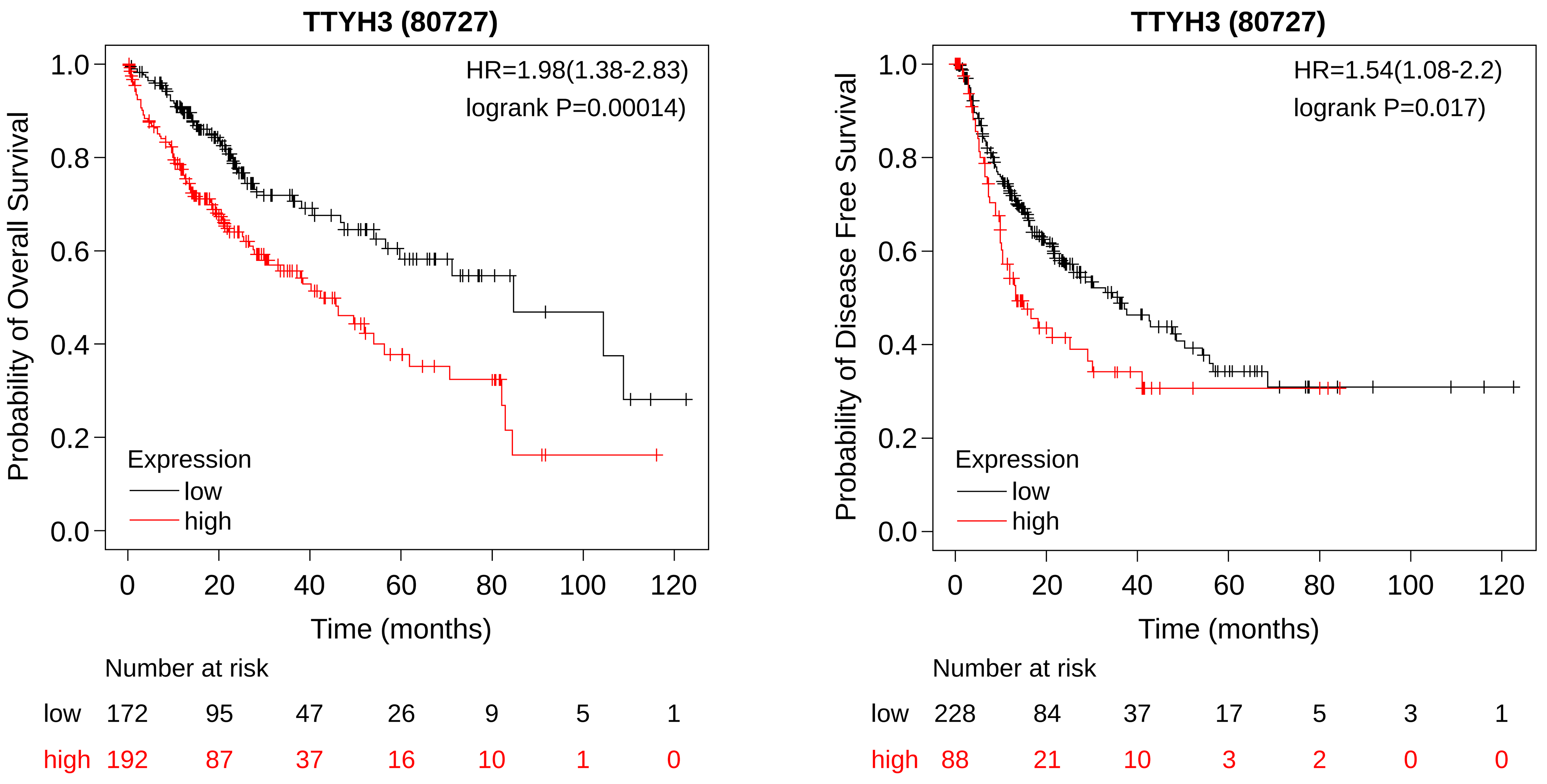

Figure S4

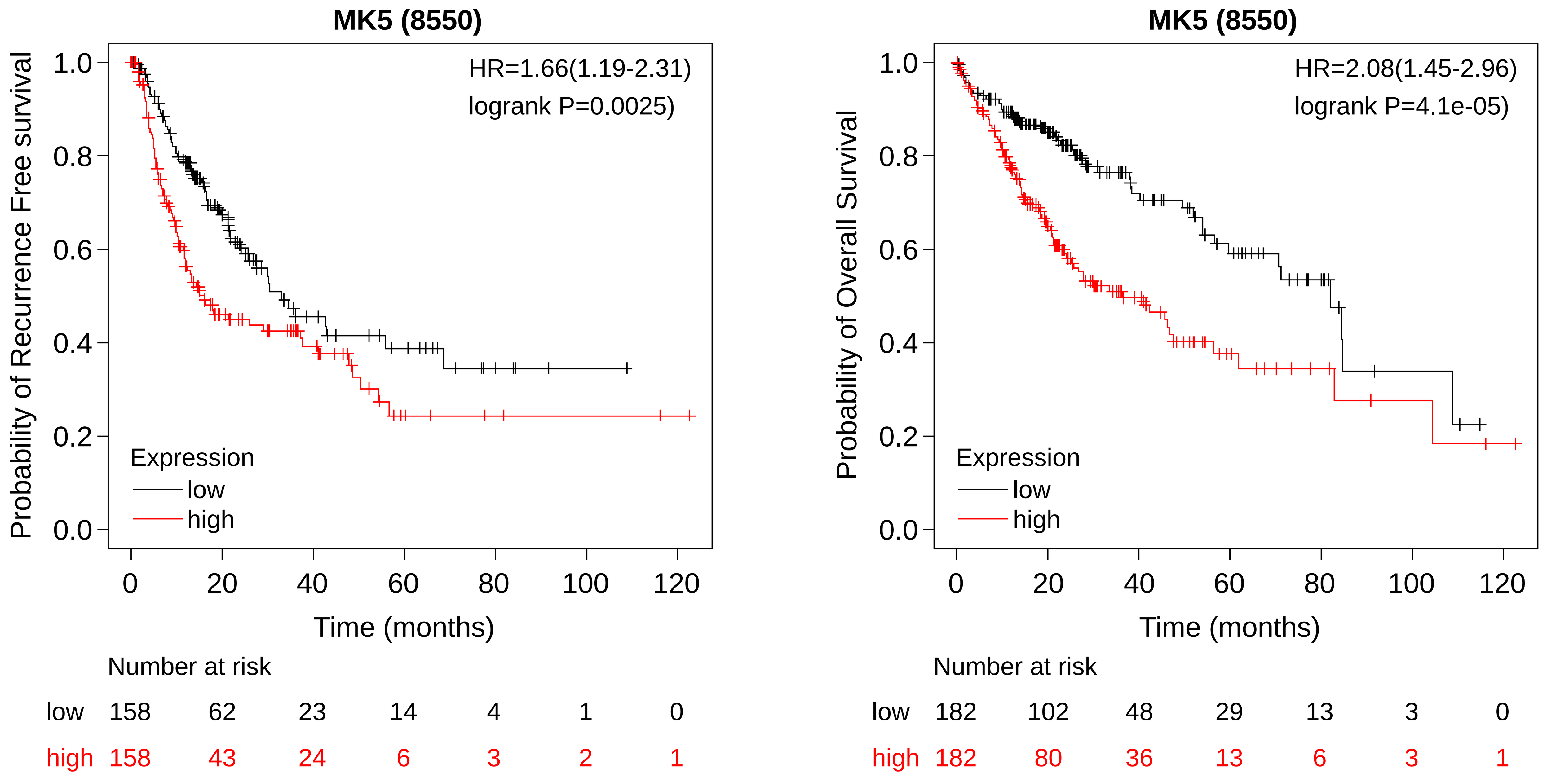

Figure S5

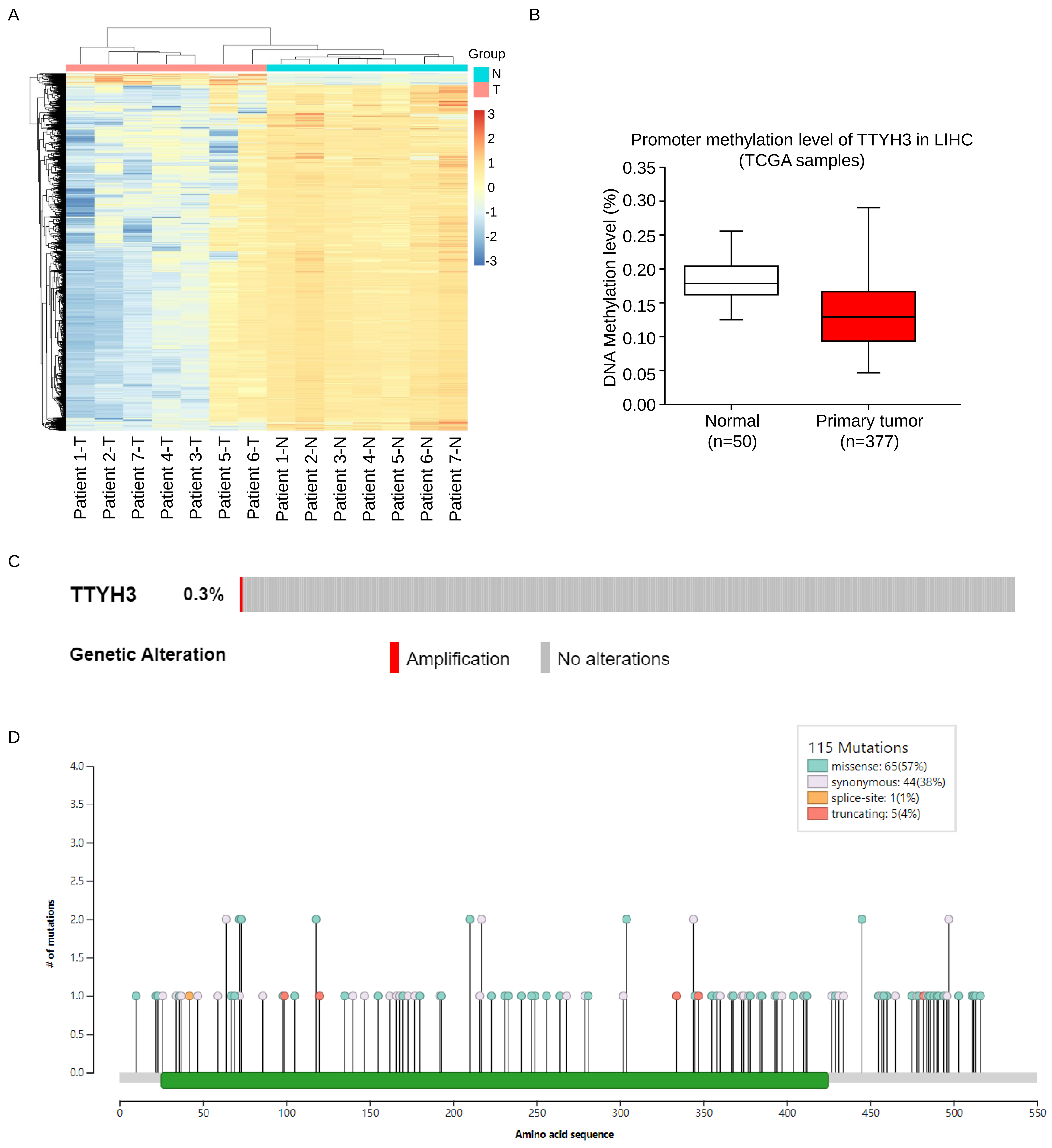

Figure S6

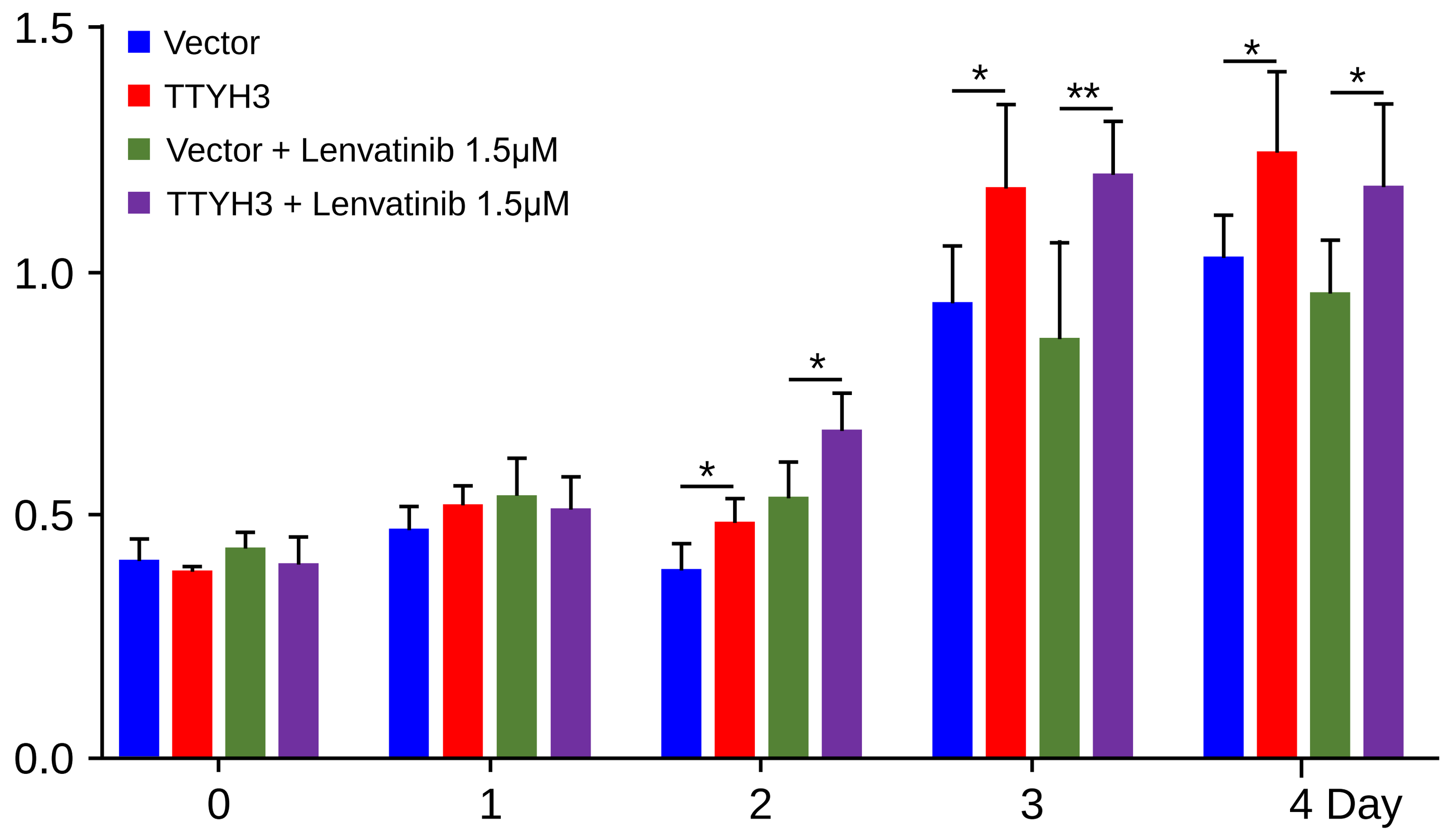

Supplemental Table S1 Univariate analysis of continuous variables.

Variable	No recurrence (n=30)	Recurrence (n=19)	p value
**Age, yr**	53.30±8.75	55.16±8.38	0.465
**Tumor size, cm**	3.75 (3.12-4.92)	4.75 (3.50-8.00)	0.001
**Neutrophils,10^9/L**	2.68 (2.54-3.98)	3.50 (1.94-6.75)	0.782
**Lymphocyte,10^9/L**	1.96±1.00	1.71±0.55	0.322
**PLT**	162.50±58.69	169.95±73.73	0.697
**INR**	0.88±0.07	0.88±0.09	0.699
**PT**	9.85 (8.79-10.40)	10.00 (7.65-11.33)	0.742
**Alb**	40.10±4.28	38.60±3.12	0.192
**Tbil**	17.21±6.48	15.52±6.45	0.376
**ALT**	32.5 (30.20-55.52)	48.50 (16.48-141.32)	0.417
**AST**	24.50 (23.05-38.50)	32.0 (9.51-103.70)	0.259
**GGT**	27.00（26.36-51.74）	51.50 (33.80-93.00)	0.050
**NLR**	1.59 (1.01-4.09)	1.63 (0.87-4.64)	0.538
**PLR**	88.13(75.98-116.20)	94.74 (81.81-135.95)	0.367

Supplemental Table S2 Univariate analysis of categorical variables.

Variable		No recurrence (n=30)	Recurrence (n=19)	*P* value
**Gender**	Female	3 (10.00)	2 (10.5)	1.00
	Male	27(90.00)	17 (89.5)	
**TTYH3 expression**	Low	21 (70.00)	4 (21.10)	0.001
	High	9 (30.00)	15 (78.90)	
**Edmondson-Steiner Grade**	I/II	6 (20.70)	8 (42.10	0.110
	III/IV	23 (79.30)	11 (57.9)	
**Liver capsule invasion**	No	13 (43.30)	7 (36.80)	0.652
	Yes	17(56.70)	12 (63.20)	
**Number of tumors**	Single	27 (90.00)	15 (78.90)	0.510
	⩾2	3 (10.00)	4 (21.10)	
**Satellite nodules**	No	28 (93.30)	15 (78.90)	0.294
	Yes	2 (6.70)	4 (21.10)	
**Cirrhosis**	No	4 (13.30)	26 (86.70)	0.444
	Yes	5 (26.30)	14 (15.50)	
**BDTT**	No	29 (96.70)	19 (100.00)	1.00
	Yes	1 (3.30)	0 (0.00)	
**MVI**	No	21 (70.00)	11 (57.90)	0.386
	Yes	9 (30.00)	8 (42.10)	
**Surgical options**	open	29 (96.70)	19 (100.00)	1.00
	laparoscope	1 (3.30)	0(0.00)	
**AFP, ng/ml**	≤400	23 (76.70)	12 (63.20)	0.308
	>400	7 (23.30)	7 (36.80)	
**HBV**	No	2 (6.70)	3 (15.80	0.587
	Yes	28 (93.30)	16 (84.20)	

Supplemental Table S3 Multivariate regression analysis

Variable	OR (95%CI)	*P* value
**TTYH3**	9.641 (1.979-46.96)	0.005
**Tumor size, cm**	1.499(1.034–2.174)	0.033

Supplemental TableS4. Detailed material information

4.1 Antibodies

**Name**	**Citation**<br>Pubmed ID	**Supplier**	**Cat no.**	**Clone no.**
anti-Bax	**33778195**<br>**34147843**<br>**34149930**	Cell Signaling Technology	#2772S	N/A
anti-Bcl-2	**34124093**<br>**34046147**<br>**34017393**	Cell Signaling Technology	#2876S	N/A
anti-N-cadherin	**34121323**<br>**33767919**<br>**34108444**	Cell Signaling Technology	#13116	N/A
anti-E-cadherin	**34142050**<br>**34158864**<br>**34103528**	Cell Signaling Technology	#3195	N/A
anti-β-catenin	**34187415**<br>**34108491**<br>**34099634**	Cell Signaling Technology	#8480	N/A
anti-phospho-GSK-3β	**33762939**<br>**33305480**<br>**33275593**	Cell Signaling Technology	#5558	N/A

anti-GSK-3β	**33931588**<br>**33139730**<br>**32444788**	Cell Signaling Technology	#12456T	N/A
anti-TTYH3	**N/A**	Abcam	#ab240580	N/A
anti-PRAK/MK5	**N/A**	Abcam	#ab93800	N/A
anti-TTYH3	**N/A**	Thermo Fisher	PA5-62800	N/A
anti-Ki-67	**34126361**<br>**33292235**<br>**33021054**	Cell Signaling Technology	#9027S	N/A
anti-GAPDH	**34146958**<br>**34132367**<br>**34183021**	Cell Signaling Technology	#5174S	N/A
anti-c-Myc	**34988408**<br>**34930894**	Cell Signaling Technology	#5605	N/A

4.2 Cell lines

**Name**	**Citation (Pubmed ID)**	**Supplier**	**Cat no.**	**Passage no.**	**Authentication test method**
HCCLM3	N/A	Shanghai Yansheng Industrial Co., Ltd.	N/A	6 after purchase	STR
Hep3B	N/A	Shanghai Genechem	N/A	5 after purchase	STR

4.3 Organisms

Name	Citation<br>PubMed ID	Supplier	Strain	Sex	Age	Overall n number
Balb/c nude mice	33416179<br>33986244<br>30572882	Beijing Vital Laboratory Animal Technology Co., Ltd.	Balb/c	male	4-6 weeks	24

4.4 Sequence based reagents

Name	Sequence	Supplier
qPCR primer: TTYH3-Forward	TGCTGAATGGCACGGAGGTGAA	bgitech
qPCR primer: TTYH3-Reverse	CTCCACGCCGTCATAGCAGAAG	bgitech
qPCR primer: GAPDH-Forward	GTCTCCTCTGACTTCAACAGCG	bgitech
qPCR primer: GAPDH-Reverse	ACCACCCTGTTGCTGTAGCCAA	bgitech

4.5 Biological samples

Description	Source	Identifier
Human liver biopsy	Department of Hepatobiliary and Pancreatic Surgery, The Affiliated Hospital of Qingdao University	Medical Ethics Committee of the Affiliated Hospital of Qingdao University.

4.6 Deposited data

**Name of repository**	**Identifier**	**Link**
The Cancer Genome Atlas (TCGA)	Hepatocellular carcinoma gene expression database by RNAseq (IlluminaHiSeq percentile, n = 423)	http://cancergenome.nih.gov/publications/publicationguidelines

4.7 Software

**Software name**	**Manufacturer**	**Version**
ImageJ	National Institutes of Health	1.8.0
Graphpad Prism	GraphPad Software	Prism 8
SPSS	IBM	SPSS 20.0

4.8 Other (e.g., drugs, proteins, vectors etc.)

Vector(wt-TTYH3) Lentivirus	Shanghai Hanbio	Cat no.: LV45070726
Overexpress wt-TTYH3 Lentivirus	Shanghai Hanbio	Cat no.: LV45070727
Control(wt-TTYH3)	Shanghai Genechem	Cat no.: P20092200
Overexpress wt-TTYH3 plasmid	Shanghai Genechem	Cat no.: GCPE0258060
Control(MK5)	Shanghai Genechem	Cat no.: P19053000
Overexpress MK5 plasmid	Shanghai Genechem	Cat no.: GOSE0210505
Control(mut-TTYH3)	Shanghai Genechem	Cat no.: P21070100
Overexpress mut-TTYH3 plasmid	Shanghai Genechem	Cat no.: GOSE0297187
Negative control siRNA	GenePharma	Cat no.: A10001

TTYH3 siRNA1	GenePharma	Cat no.: A10001
TTYH3 siRNA2	GenePharma	Cat no.: A10001
Negative control siRNA	GenePharma	Cat no.: A10001
MK5 siRNA1	GenePharma	Cat no.: A10001
MK5 siRNA2	GenePharma	Cat no.: A10001
Cacl2	Solarbio	Cat no.: G0071
BAPTA	Medchemexpress	Cat no.: HY-100168

4.9 Please provide the details of the corresponding methods author for the manuscript:

Chengzhan Zhu, MD
Department of Hepatobiliary and Pancreatic Surgery
The Affiliated Hospital of Qingdao University
No.16 Jiangsu Road, Qingdao
China.
Tel:+8618661806921 ; Fax: +8653282911885

Email: zhuchengz@qduhospital.cn
